# Genome-Wide Identification and Characterization of Salinity Stress-Responsive miRNAs in Wild Emmer Wheat (*Triticum turgidum* ssp. *dicoccoides*)

**DOI:** 10.3390/genes8060156

**Published:** 2017-06-06

**Authors:** Kewei Feng, Xiaojun Nie, Licao Cui, Pingchuan Deng, Mengxing Wang, Weining Song

**Affiliations:** 1 State Key Laboratory of Crop Stress Biology in Arid Areas, College of Agronomy and Yangling Branch of China Wheat Improvement Center, Northwest A&F University, Yangling 712100, Shaanxi, China; fkwyc@hotmail.com (K.F.); juelianjunjie@foxmail.com (L.C.); 519wangsui@163.com (P.D.); machine@nwsuaf.edu.cn (M.W.); 2 Australia-China Joint Research Centre for Abiotic and Biotic Stress Management in Agriculture, Horticulture and Forestry, Yangling 712100, Shaanxi, China

**Keywords:** salinity stress, wild emmer, microRNAs (miRNA), qRT-PCR, deep sequencing

## Abstract

MicroRNAs (miRNAs) are a class of endogenous small noncoding RNAs which regulate diverse molecular and biochemical processes at a post-transcriptional level in plants. As the ancestor of domesticated wheat, wild emmer wheat (*Triticum turgidum* ssp. *dicoccoides*) has great genetic potential for wheat improvement. However, little is known about miRNAs and their functions on salinity stress in wild emmer. To obtain more information on miRNAs in wild emmer, we systematically investigated and characterized the salinity-responsive miRNAs using deep sequencing technology. A total of 88 conserved and 124 novel miRNAs were identified, of which 50 were proven to be salinity-responsive miRNAs, with 32 significantly up-regulated and 18 down-regulated. miR172b and miR1120a, as well as mi393a, were the most significantly differently expressed. Targets of these miRNAs were computationally predicted, then Gene Ontology (GO) and Kyoto Encyclopedia of Genes and Genomes (KEGG) analysis found that the targets of salinity-responsive miRNAs were enriched in transcription factors and stress-related proteins. Finally, we investigated the expression profiles of seven miRNAs ranging between salt-tolerant and sensitive genotypes, and found that they played critical roles in salinity tolerance in wild emmer. Our results systematically identified the salinity-responsive miRNAs in wild emmer, not only enriching the miRNA resource but also laying the foundation for further study on the biological functions and evolution of miRNAs in wild wheat and beyond.

## 1. Introduction

Wheat is one of the most important crops around the world, with about 620 Mt of production annually, and it provides more than 20% of total human food calories [1]. However, abiotic stresses such as drought and salinity shed a significantly negative impact on wheat production and these negative effects are gradually increasing due to declining water supply, land degradation and climate change [2]. Due to the narrowed genetic diversity of cultivated wheat, more and more breeding programs have focused on the utilization of wild relatives with large genetic potentiality to improve wheat tolerance to abiotic stresses [3]. Wild emmer wheat (*Triticum turgidum* ssp. *dicoccoides*) is the progenitor of cultivated wheat, which adapts to a broad range of environments with rich genetic alleles related to abiotic stress [4]. Some genotypes of wild emmer were found to be highly tolerant to salt stress and could be helpful for wheat improvement [3,5].

Salinity is regarded as a major abiotic stress factor worldwide, which limits plant growth and development by eliciting sodium toxicity and impairing ionic and osmotic homeostasis [6]. Uncovering the molecular mechanism of salinity response in crops holds promise for meeting the challenges of food security and global climate change [7]. Extensive studies have been conducted to identify and characterize the genes and regulators involved in the biological process and the metabolic network of salinity stress response, which provided some clues for the mechanism as well as the resource for genetic improvement of crop salt tolerance [8]. However, less is known about the regulatory network controlling the salt response and tolerance.

MicroRNAs (miRNAs) are a class of small (18–24 nt), non-coding RNAs that function as important negative regulators to gene expression through post-transcriptional degradation or translational repression of their target mRNAs [9,10]. Many studies have demonstrated that miRNAs play indispensable roles in plant growth and development as well as in diverse stress responses such as to heat [11], drought [12] and other stressors [13,14]. For salinity stress, previous studies showed that miRNAs were in the hub of the regulatory network in response to salinity stress [15,16,17]. The salinity stress-related miRNAs and their targets have been identified in many crops such as maize [18], rice [17], cotton [19], barley [7] and bread wheat [20]. miR156, miR169, miR396, etc., proved to be differentially expressed miRNAs under salinity stress and showed a different expression profile in different species, suggesting they might mediate gene regulation under salinity stress with species-specific patterns [7]. Many miRNA families, such as Tae-miR171, Tae-miR393, and Tae-miR855, as well as Tae-miR408, were reported to have induced expression under salinity stress in wheat [20,21].

Although numerous stress-related miRNAs have been identified and analyzed in diverse plant species, only drought-responsive miRNAs have been reported in wild emmer wheat based on a microarray [22] or a homology-based prediction approach [23]. High throughout sequencing for identification miRNAs in wild emmer has not been performed up to now. Here, to gain insight into the role of miRNAs playing in salinity stress tolerance in wild emmer wheat, we identified and characterized the salinity responsive miRNAs through high-throughput sequencing combined with bioinformatic analysis. Our research will provide a basis for investigation of miRNA-modulating pathway underlying salt stress response in wild emmer and the identified salt-responsive miRNAs will serve as a key resource in further wheat improvement and beyond.

## 2. Materials and Methods

### 2.1. Plant Materials and Salt Treatment

The wild emmer accession B5, a high salinity tolerant genotype obtained from our previous work, was used in this study. Seeds were sterilized and germinated in vermiculite at 28 °C. Then, healthy germinated plants were selected uniformly and transplanted into hydroponic container with half-strength of modified Hoagland’s solution. The experiment was carried out in a growth chamber (ZPG-400B, Dong Tuo, Heilongjiang, China) at 30/20 °C (day/night), a relative humidity of 55–65% and a photoperiod of 14 h light (6 model)/10 h dark (0 model). At the three-leaf stage, the stress treatment began by adding NaCl to the growth medium with 50 mmol/L increments every day, until reaching the final concentration of 150 mmol/L NaCl. The control treatment was set using the normal solution without NaCl. Salinity-treated and control whole plants were harvested at 24 h after the final concentration of 150 mM for the downstream experiments. Two biological replicates were used for small RNA sequencing.

### 2.2. Small RNA Library Construction and Sequencing

Total RNA was extracted using TRIzol reagent (TaKaRa Co., Tokyo, Japan) according to the manufacturer’s instructions. RNA purity was checked using the NanoPhotometer spectrophotometer (IMPLEN, Westlake Village, CA, USA). RNA isolated from root and leaf tissues was mixed in equal amounts for further RNA sequencing. Small RNAs were ligated sequentially to 5′ and 3′ RNA/DNA chimeric oligonucleotide adaptors (Illumina), and the resulting ligation products were gel purified by 10% denaturing PAGE and reverse-transcribed to produce cDNAs. The cDNAs were sequenced using a Genome Analyzer IIx System (CapitalBio Technology Inc., Beijing, China).

### 2.3. miRNA Identification

Individual sequence reads with the base quality scores were produced by the Illumina/Solex platform. Adapter sequences and low quality reads were filtered firstly. All identical sequences were counted and eliminated from the initial data set. The resulting set of the unique sequences with associated read counts is referred to as the sequence tags. The unique reads were mapped onto the *Triticum durum* genome (https://wheat-urgi.versailles.inra.fr/Seq-Repository/Assemblies) using the program Bowtie [24]. Unique reads were screened against the Rfam database (Release 12.2) [25] using Bowtie for small RNA (ribosomal (rRNA), transfer RNA (tRNA) and small nucleolar RNA (snoRNA), etc.) annotation, and the annotated reads were removed. The remaining small RNA reads were used for BLAST against all of the mature plant miRNAs deposited in miRbase release 21 [26] using Patscan [27] to identify conserved miRNAs. The criteria to define potential conserved miRNAs is no more than 2 mismatches. For the novel miRNA identification, to avoid repeated prediction and reduce the calculation amount, the pre-mature microRNA genomic locus was extracted if the distance of the candidate unique reads in the reference genome was less than 200 bp. For each pre-mature miRNA in the genome, the 200 nt up and down-stream sequences were extracted for secondary structure analysis. The software Einverted of Emboss [28] was used to find the inverted repeats (stem loops or hairpin structure), with the parameter threshold = 40, match score = 3, mismatch score = −3, gap penalty = 6, and maximum repeat length = 240 [29]. Each inverted repeat was extended 10 nt on each side; the secondary structure of the inverted repeat was predicted by RNAfold [30]. Unique reads in the inverted repeats were evaluated by MirCheck using modified parameters as described by Wu et al. [29]. Finally, precursors (hairpins) of microRNAs obtained from MirCheck analysis were checked manually to remove false predictions.

### 2.4. Detection of Differentially Expressed miRNAs

All the identified conserved and novel miRNAs were used to detect differentially expressed miRNAs. miRNA count was normalized as transcripts per million (TPM) with the following formula: normalized expression = mapped read count/total reads × 10^6^. The software edgeR [31] was used to identify microRNAs showing statistically significant differences in relative abundance (as reflected by total count of individual sequence reads) between the two types of small RNA libraries. Finally, microRNAs with a *p* value ≤ 0.05 were marked to be significantly different between normal and stressed samples.

### 2.5. Target Gene Prediction

All identified miRNA sequences were used to query the emmer wheat cDNA (ftp://ftpmips.helmholtzmuenchen.de/plants/wheat/IWGSC/IWGSC_genePredictions_of_other_wheat_species/) for potential target sequences using the Patscan tool with the following default parameters: 3 mismatches, 0 insertions, and 0 deletions. Only hits that had no mismatches in positions 10 and 11 in the mature miRNAs were considered to be candidate targets [32]. Gene Ontology (GO) enrichment and Kyoto Encyclopedia of Genes and Genomes (KEGG) pathway analysis of these targets were performed based on the GO (http://www.geneontology) and KEGG (http://www.genome.jp/kegg/database) databases, respectively.

### 2.6. Validation of the Expression of miRNAs by qRT-PCR Analysis 

To validate the identified miRNAs, the stem-loop real-time PCR method [33] was used firstly. The PCR system of 20 μL volume contained 1 μL DNase I-treated RNA, 0.5 μL dNTP (deoxy-ribonucleoside triphosphate) mix (10 mM), 0.25 μL SuperScript III Reverse Transcriptase (Invitrogen, Grand Island, NY, USA), 4 μL 5× first-strand buffer, 2 μL DTT (0.1 M), 0.1 μL RNase inhibitor-HPRI (Takara, Dalian, China, 40 units/μL), 11.15 μL nuclease-free water and 1 μL stem-loop RT primer for each miRNA (1 μM). 

Furthermore, the expression patterns of seven identified salinity-responsive miRNAs were investigated using the A-tail qRT-PCR method, including three highly conserved stress-related miRNAs and the four most significantly differentially expressed novel miRNAs. The salt-tolerant genotype B5, together with the sensitive genotype GilbourA9, were used and treated with two salinity stress levels (150 mmol/L and 250 mmol/L NaCl). Leaf samples were harvested at 0 h, 3 h, 6 h, 12 h, and 24 h after treatment for RNA isolation. The One Step Primescript miRNA cDNA Synthesis Kit (TianGen Inc., Beijing, China) was used to synthesize the cDNAs, which were then amplified with a mature miRNA sequence in combination with the universal adaptor as the primer for miRNA expression analysis. Real-time PCR analysis was carried out on an ABI StepOnePlus System as follows: 2 min at 94 °C followed by 34 cycles of 20 s at 94 °C, then 34 s at 60 °C. The amplification specificity was monitored by melting curve after PCR. The qRT-PCR was performed in three replications with the 18S rRNA as a reference gene.

The 2^−ΔΔCT^ method (CTmiRNA − CT18S rRNA) and ΔΔCT = (ΔCT treatment − ΔCT control) was used to evaluate gene expression [34]. Student’s *t*-test was used as a statistical tool for the analysis of differences in expression among the triplicates (*n* = 3, *p* < 0.05). The primers used in this study are listed in Table S3.

## 3. Results

### 3.1. Small RNA Sequencing

A total of four small RNA (sRNA) libraries were constructed with two biological replicates for both salt-stress and control treatments. The reads statistics generated by Illumina (San Diego, CA, USA) sequencing are shown in Table 1. The total raw reads of the salinity stress and the control were 34,461,291 and 33,039,140, respectively. The raw sequence data reported in this paper have been deposited in the Genome Sequence Archive [35] at the BIG Data Center [36], Beijing Institute of Genomics (BIG), Chinese Academy of Sciences, under accession number PRJCA000426. After discarding the 5′ and 3′ adapters, polluted reads and reads smaller than 18 nt, 28,557,077 (82.87%), 28,222,085 (85.42%) high quality reads were retained, representing 5,580,879 and 4,565,334 unique reads in the salinity stress and the control samples, respectively (Table 1). For the total sRNA reads, in the majority the length was 24 nt in the salinity stress data and 22 nt in the control. Of the unique reads, the most abundant was 24 nt in both salinity stress data and control (Figure S1). The total reads numbers of salinity stress and control mapped to the *T. turgidum* genome were 17,081,214 (59%) and 11,269,732 (40%), respectively. The sRNA reads were then annotated by the RFam database and miRbase v21.0 (http: //www.mirbase.org/) for classification. Potential miRNA reads, tRNA, rRNA, snoRNA, and other sRNAs were also identified (Table 2).

### 3.2. Identification and Characterization of Salinity-Responsive miRNAs

Based on the method as described above, a total of 88 conserved miRNAs, belonging to 41 miRNA families, were detected in this study. miR-166a was the most abundant miRNA with a total of 83,867 times detected while 10 miRNAs such as miR-1120b and miR-5181 were regarded as the least accumulated miRNAs as they were detected only once (Table S1). Meanwhile, a total of 134 novel miRNAs were also identified. Novel-41 was the most accumulated miRNA with 2650 detection times. For the confirmation of hairpin structures, all the precursors of these miRNAs possessed the typical stem-loop structures (Figure S1 and Figure S3). Previous studies reported that base composition can affect the physiochemical properties and the secondary structures of miRNAs [37]. Analysis of the nucleotide bias in the total miRNAs of wild emmer wheat showed the first and 24th nucleotide position preferred U (Figure S2A). For miRNAs of different lengths, the first nucleotide was primarily U for 20 nt, 21 nt and 22 nt, G for 18 nt and A for 24 nt (Figure S2B).

**Figure 1:**
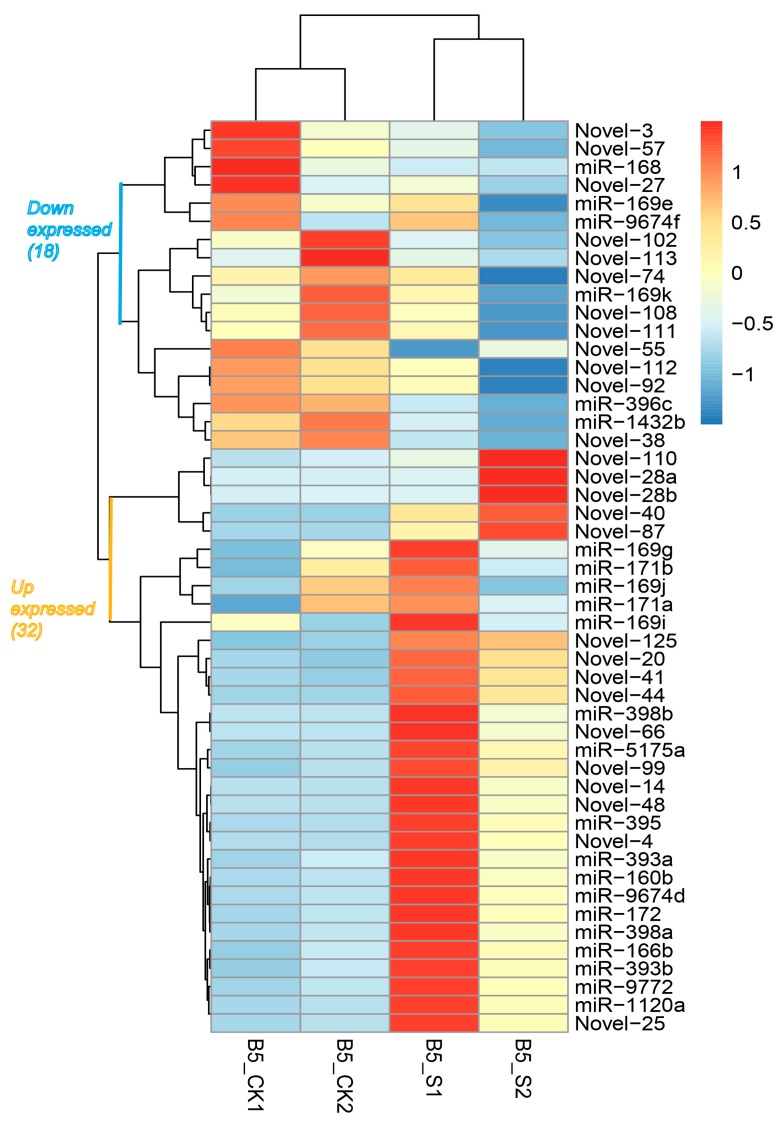
Heatmap of different expressed micro RNAs (miRNAs) of wild emmer under salinity stress based on scaled normalization counts (TPM, transcripts per million) values.

Of all the miRNAs identified in wild emmer, 23 conserved and 27 novel miRNAs were marked to be significantly (*p*-value < 0.05) differentially expressed between salinity stress and the control (Table 3, Figure 1). A series of previously reported salinity-responsive miRNAs including miR160, miR169, miR171 were also identified in our study. Further analysis revealed that 17 of the 23 conserved miRNAs were up-regulated under salinity stress compared to in normal conditions, while the other six were down-regulated. Among the up-regulated miRNAs, the miR393 family was observed to be the highest up-regulated, with a value 10.64 times higher than that of normal conditions. For the six down-regulated conserved miRNAs, miR168 showed the highest down-regulation. Among the 27 differentially expressed novel miRNAs, 15 up-regulated and 12 down-regulated miRNAs were found in salinity samples compared to control. The novel-25, novel-41 and the novel-28a miRNAs showed more than ten times the variation in expression under salinity stress compared to normal conditions.

### 3.3. Target Gene Prediction and Annotation 

In order to better understand the potential biological function of the miRNAs identified, we predicted target genes of miRNAs using the available emmer wheat cDNA resource. A total of 5422 putative target genes were obtained (conserved miRNA: 3830, novel miRNA: 2908), with an average of 19 targets for every miRNA. The number of targets of each miRNA varied from one to 693 and one to 533 for conserved and novel miRNAs, respectively. miR-5205 was detected with the largest number of targets with the value of 744. Among the 280 miRNAs, 107 (38.2%) miRNAs (conserved: 51, novel: 56) were found with more than 10 predicted targets (Table S2), while 48 miRNAs were found to have only one target. All putative target genes were analyzed by GO analysis and categorized by molecular function (59.90%), biological process (29.70%), and cellular component (10.40%), respectively. For molecular function categories, genes were mainly involved in protein binding (10.09%) and ATP binding (8.98%). For biological processes, protein phosphorylation (10.07%), the oxidation-reduction process (9.44%) and the metabolic process (6.70%) were highlighted. As for the cellular component, it was mainly localized in the nucleus (20.59%), membrane (20.34%) and integral component of membrane (15.93%) (Table S4). The KEGG analysis revealed that miRNAs were mainly involved in different metabolisms (46.95%) and genetic information processing pathways (32.93%) (Table S5). 

**Figure 2:**
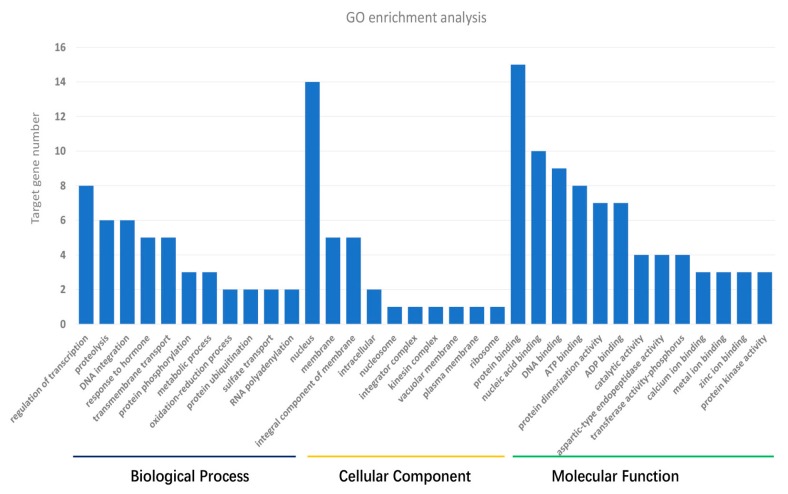
Gene Ontology (GO) enrichment analysis of target genes of the differentially expressed miRNAs.

For the targets of the salinity stress-responsive miRNAs, the GO enrichment showed that they were mainly involved in molecular function (56.36%) and the biological process (30.08%), and only 13.56% were involved in the cellular component (Figure 2, Table S6). The main processes of target genes involving in were binding of molecules (protein binding, ATP binding, ADP binding and some metal ion binding) in the category of molecular function. Another important part in molecular function could be summarized as related biochemical activity such as transferase activity, transmembrane transporter activity and potassium ion or sodium proton transporter activity. For the biological process, regulation of transcription, proteolysis, transmembrane transport, and response to hormones were identified as processes with multiple target genes. However, many target genes were annotated in molecule or ion transport such as for sodium ion, potassium ion, sulfate or cation transport and response to salt stress or oxidation-reduction process. Further comparison analysis indicated target genes of up-regulated miRNAs showed higher proportions in the biological process category (34.59%) and lower proportions (50.94%) in the molecular function category than for target genes of down-regulated miRNAs (20.78% and 67.53% respectively). The KEGG pathway analysis also revealed different patterns between up-regulated and down-regulated miRNAs. In the metabolism category, a total of nine pathways related to carbohydrate, glycan and amino acid metabolism were involved in the up-regulated miRNAs, while only one pathway about oxidative phosphorylation was found in target genes of down-regulated miRNAs. In the genetic information category, four pathways related to folding, sorting and degradation, and three pathways of replication and repair were found to be enriched in targets of down-regulated miRNAs, while targets of up-regulated miRNAs were mainly in transcription and translation. In environmental information processing, targets of down-regulated miRNAs were annotated mainly related to membrane transport, while those of up-regulated miRNAs were annotated as related to signal transduction (Table S7).

These results suggest that miRNAs may play important roles in signal transduction and ion homeostasis under salt stress in wild emmer. The miRNA involved in salt-related terms of GO and KEGG, such as response to hormone stimulus, response to endogenous stimulus, and small GTPase-mediated signal transduction could provide potential candidates for further functional studies.

### 3.4. qRT-PCR Analysis of Salinity-Related miRNAs

**Figure 3:**
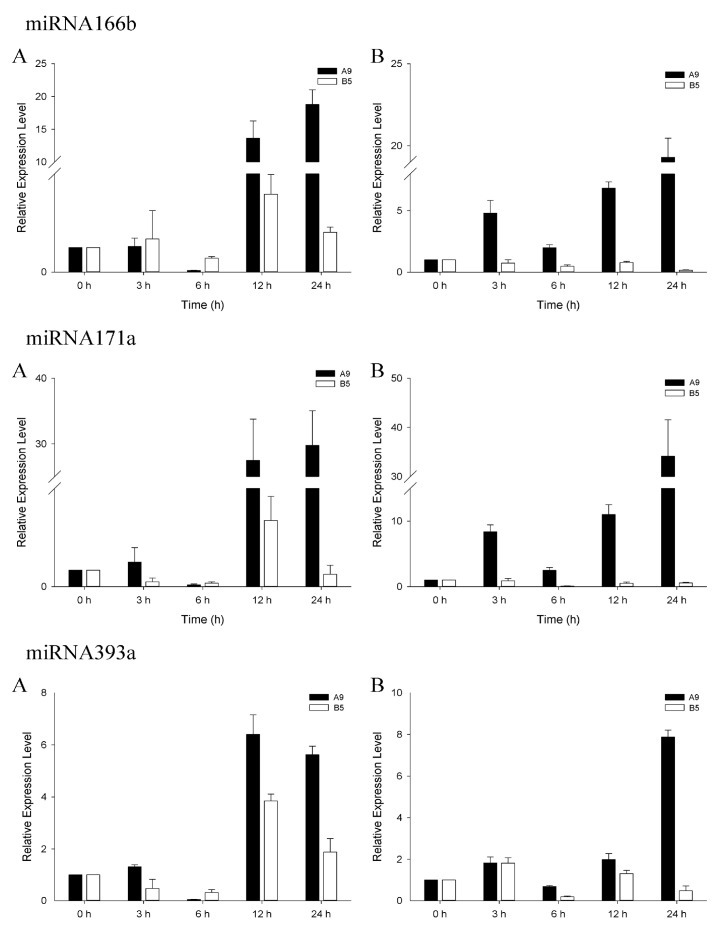
Relative expression of three conserved salinity stress responsive miRNAs of wild emmer genotypes A9 (sensitive, black bars) and B5 (tolerant, white bars). (**A**): 150 mM; (**B**): 250 mM. Values are the mean ± standard errors (*n* = 3). miRNA copy numbers were normalized by comparison with wheat 18S ribosomal RNA (rRNA); individual miRNA expression levels were then normalized by comparison with their expression in the control treatment, which was set to 1.0.

**Figure 4:**
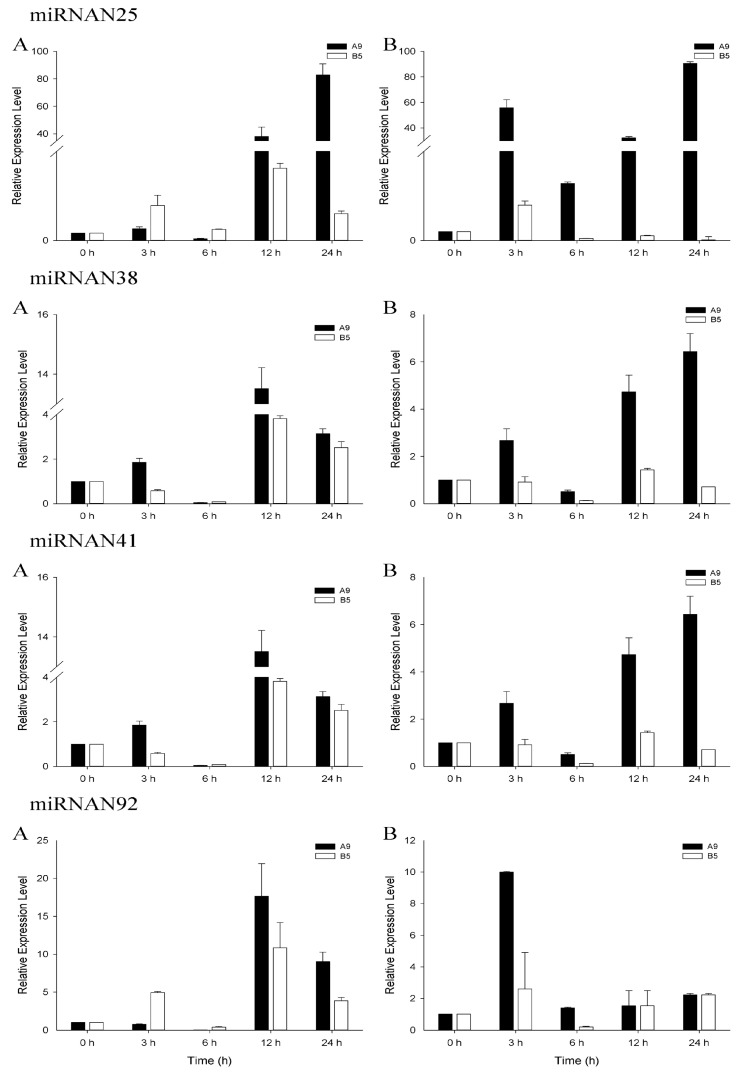
Relative expression of four novel salinity stress-responsive miRNAs of wild emmer genotypes A9 (sensitive, black bars) and B5 (tolerant, white bars). (**A**): 150 mM; (**B**): 250 mM. Values are the mean ± standard error (*n* = 3). miRNA copy numbers were normalized by comparison with wheat 18S rRNA; individual miRNA expression levels were then normalized by comparison with their expression in the control treatment, which was set to 1.0.

Stem-loop qRT-PCR was performed using the same RNA samples (for sequencing) to validate the sequencing and identification results. All the miRNAs validated by stem-loop RT-PCR displayed consistent expression patterns with the result of sequencing (Figure S4). We performed qRT-PCR analysis to further investigate and compare the expression patterns of salinity-responsive miRNAs in the salinity-sensitive genotype A9 and tolerant genotype B5. To comprehensively plot the expression profiles, we performed the experiment with two concentrations of NaCl (150 mM, 250 mM) and five time courses (0 h, 3 h, 6 h, 12 h, 24 h). Three highly conserved (miR166b, miR171a and miR393a) and four novel (miRN25, miRN38, miRN41 and miRN92) salinity stress-responsive miRNAs were analyzed. Generally, the expression trends of these miRNAs obtained from qRT-PCR were consistent with that of sequencing (Figure 3 and Figure 4). However, miRN38 and miRN92 showed some differences, which may due to the PCR analysis using a leaf while the sequencing sample was from the whole plant. Besides, the physiological differences among samples or the high fluctuation in the expression of some miRNAs in plants in response to stress may also cause the different results between qRT-PCR and the sequencing [38,39]. The dynamic changes in the expression profiles of the miRNA response to salt stress were observed (Figure 3 and Figure 4). For tolerant genotype B5 under the 150 mM NaCl condition, the seven miRNAs shared the similar expression patterns which decreased at the early stages (3 h, 6 h) with the lowest expression at 6 h, followed by up-regulation at 12 h and 24 h. Under the 250 mM NaCl condition, the expression patterns of miRNAs were rather different from those at 150 mM. Most of them showed a relatively down-regulation compared to the normal condition except for three miRNAs up-regulated at 3 h including miR393a, miRN25, and miRN92. For the salinity-sensitive genotype A9, when under the 150 mM NaCl condition, the expression pattern was almost consistent with that of tolerant genotype B5. All the seven miRNAs a showed relatively lower expression level at 6 h and the highest expression level at 12 h, except for miR171a and miRN25 which showed the highest expression level at 24 h. While under the 250 mM condition, six of the seven miRNAs shared a similar expression pattern, except for miRN92. Generally, the relatively expression level of these miRNA showed the lowest level at 6 h and reached the peak at 24 h. While for miRN92, the highest expression level occurred at 3 h and kept almost equal at other time courses (Figure 4).

## 4. Discussion

### 4.1. Genome Wide Identification of miRNAs in Wild Emmer Using High-Throughput Sequencing

Environmental abiotic stresses such as salinity harmfully affect the development and growth of plants. For wheat, salinity is a major abiotic stress factor which significantly reduces wheat yields worldwide. With high genetic diversity, wild emmer wheat (*T. turgidum* ssp. *dicoccoides*) presents a rich gene pool for wheat breeding programs. In recent years, many studies have suggested that a lot of genes are involved in response to salinity stress and exhibit different patterns of expression which may be regulated at the post-transcriptional level by miRNA in plants. The salinity-related molecular mechanisms, including miRNA-regulated pathways of wheat and its related species, are crucial to developing high yielding wheat varieties in high salinity areas. For wild emmer wheat, although it was considered as a vital genetic resource for salinity tolerant improvement, no systematic identification of miRNAs by sequencing has been reported till now. High-throughput sequencing is an effective method for miRNA discovery and miRNA expression profiling analysis and has been widely applied to miRNA research [37]. The technology is able to capture extensive collections of genome-wide or transcriptome-wide miRNAs [9]. Kantar et al. [22] reported miRNAs expression patterns responsive to drought stress based on microarray analysis. Although amounts of miRNAs (205 miRNAs in control and 438 miRNAs in drought stress) were detected [22], none of the novel miRNAs of wild emmer were identified as wheat-specific because of technological principle of microarray, which is based on homologous probes and showed effectiveness only on screen-known miRNAs. Akpinar et al. [23] reported 38 miRNAs by prediction from two of the root transcriptomes data (control and drought) of wild emmer and found several drought stress-related miRNAs. The data was root-specific and the homology-based prediction method was limited to its data set. In this study, we firstly obtained the miRNAome and systematically analyzed miRNAs responsive to salinity stress in wild emmer by combination of high-throughput Illumina sequencing and bioinformatic analysis. A total of 222 miRNAs, including 134 novel miRNAs, were identified. These miRNAs were proven to be of high confidence due to the combination of the deep sequencing and the computational verification. Our result greatly enriched the miRNA information of wild emmer wheat.

### 4.2. Salinity Stress-Responsive miRNAs and Their Targets in Wild Emmer

miRNAs have emerged as a potential genetic tool for understanding stress tolerance at the molecular level and eventually regulating stress response in crops [38]. The identification of salinity stress-responsive miRNAs and the functional analysis that followed assist in explaining the stress-responsive mechanism in plants. miR156, miR169, miR160, miR159, miR168, miR171, miR172, miR393 and miR396 were the most well-known salinity stress responsive miRNAs in plants summarized from previous studies in maize [18], rice [17], wheat [20,39], barley [7] and sugarcane [40]. In wild emmer wheat, we identified 23 conserved miRNAs responsive to salinity stress (Table 3). The salinity stress-responsive miRNAs mentioned above were included in our list, which indicated the existence of common key salinity stress-related miRNAs and consistent pathways responsive to salinity stress. In addition, some other conserved miRNAs and a mass of novel miRNAs were also identified as salinity-responsive in wild emmer (Table 3). Our results provide novel information about the salinity stress-responsive miRNAs of plants. Response to salinity stress in crops is comprised of a broad spectrum of processes, such as signal transduction, transcription, membrane trafficking, protein biosynthesis, etc. The GO terms of the putative targets were involved in nucleic acid binding (transcription factors) and catalytic activities regulating the development and response to abiotic stresses (Figure 2, Table S6), thereby providing useful information regarding their regulatory roles in plant physiological processes like defense and signaling. Through the KEGG analysis (Table S7), some targets of salinity response miRNAs in wild emmer were mapped to salt stress-related pathways, such as plant hormone signal transduction, flavonoid biosynthesis, ubiquitin-mediated proteolysis, apoptosis, ABC transporter, peroxisome and DNA repair [41].

It is well known that miRNA mediated the complex regulatory networks to control gene expression at the post-transcriptional level in plants [9]. The up- and down-regulation of large numbers of miRNAs induce the more dramatic expression change of multiple genes downstream. Although different plant species may cope with stress using different miRNA-mediated regulatory strategies [42], some reported hub miRNAs, such as miR171, miR169, miR393 miR396, miR398 and miR1120, etc., were associated with multiple abiotic stresses like salinity, drought [43,44], cold [45] and boron [46] or biotic stresses like fungi [47,48]. Their targets were found to be involved in sensing stress, signal transduction, etc. miR171 and miR393 were found to be up-regulated under salinity stress in wheat, barley [7] and Arabidopsis [49,50]. In our results, each of the two miRNAs belonging to the miR393 and miR171 families showed up-expression under salinity stress, especially miR393a and miR393b, about 10 times higher compared to control. The results suggested common regulatory mechanisms for salinity stress response in plants and these miRNAs may regulate the same targets in different crops [21]. The target of miR393 was a family of F-box protein genes such as *TIR1* and *AFB2* in *Arabidopsis* and rice which inhibited the lateral root growth under abscisic acid (ABA) treatment or osmotic stress [47,49]. In our study, the KEGG analysis showed that one of the targets of miR393a was the receptor protein TIR1, which regulates the hormone signal transduction pathway in wild emmer, which was similar to *Arabidopsis* and rice. GO analysis indicated that the targets of miR393a and miR393b were involved in protein and ATP binding, and enzyme activity of transferase or protein kinase which affected many molecular functions (Tables S6 and S7), which was in quite agreement with previous findings. Gupta et al. [20] found that miR168 was down-regulated while miR172 was up-regulated under salinity stress in bread wheat and the targets were mainly involved in signal transduction, development, and stress response. This was also consistent with our results of miRNA regulation (Figure 1) and target annotation (Table S6).

In wild emmer, miR166, miR171, miR398, miR396 and miR1432 were also identified as responsive to drought [22] (Table 4), which indicated these miRNAs might play key roles in both salt or drought stress-regulating pathways in wild emmer wheat. Alptekin et al. [2] suggested that both drought and salt affect the osmotic balance of plant cell. miR171 targets the myeloblastosis (MYB) family of transcription factors, which might play a role in the regulation of osmotic balance under both salinity and drought stress. GO analysis of the target genes of these miRNAs in our results found their involvement in calcium ion binding, calcium ion transmembrane transport, metal ion binding, etc., categories considered to be related to osmotic regulation (Table S6). miR396 was found to be down-regulated in both salinity and drought stress of wild emmer. Kantar et al. [22] reported that the target of miR396 was the growth factor-like (GRL) transcription factor and its putative heat-shock protein predicted by expressed sequence tags (EST) was up-regulated correlating with the down-regulation of miR396 under stresses. The heat-shock protein protects other proteins from degradation under stresses. Therefore, the down-regulation of the miR396 and the following regulation of its targets would enhance the tolerance of wild emmer in response to both drought and salinity stresses.

Furthermore, the salt- and drought-responsive miRNAs in wild emmer and related species were compared (Table 4). miRNAs such as miR156, miR171 and miR396 etc. were shown as both drought-and salt-responsive miRNAs in almost all of the five species except *Aegilops tauschii*, and miR156 was induced to express under both of the stresses in these species, indicating that the conserved drought-and salt-responsive mechanism might exist among *Triticeae* species. miR528 and miR1881 were only found to be down-regulated under drought stress, while miR393 and miR5094 were found to be up-regulated under salinity stress, indicating that these miRNAs might play a conserved role in response to the given stress. Moreover, some stress-responsive miRNAs were shown to be species-specific, such as miR1432, miR474 etc. in *T. dicoccoides,* and miR3170, miR529 etc. in *Triticum aestivum.* The listed miRNAs provided valuable information for better understanding the drought and salt stress-responsive transcriptional and post-transcription regulations in *Triticeae.*

### 4.3. miRNAs Play Critical Roles in Salinity Tolerance of Wild Emmer

miRNAs in salt stress responses are extensively based on expression profiling in plant species with varying sensitivities to salinity under variable salt levels [38]. Understanding the mechanisms of salinity response at the molecular and cellular levels between different tolerant genotypes will help us devise strategies to improve salt tolerance in crop selection programs. We performed a set of qRT-PCR analyses to determine expression patterns of seven miRNAs under different salinity stress levels and time courses in both tolerant and sensitive genotypes of wild emmer. The results revealed that the expression level of the salt-responsive miRNAs showed dynamic changes with the time and salt treatment. 

In this study, although some expression trends kept consistent with each other, a significant difference was observed, especially in the expression levels between the tolerant genotype B5 and the sensitive one, A9. miRNAs generally showed higher expression levels in A9 than B5, and miR166b, miR171a, and miRN25 were even shown several or dozens of times (Figure 3 and Figure 4). Ding et al. [18] compared miRNAs expression patterns between salt tolerant and sensitive maize and found the genotype-specific expression model of miRNAs. The similarly regulated miRNAs profiles and degrees may represent the fundamental mechanism of adapting to salt shock, and the difference might explain the distinct salt sensitivities [18]. Ganie et al. [55] reported significantly different expression patterns of osa-miR393a and its targets between two different rice genotypes. The expression of osa-mi393a in salinity-sensitive genotype tremendously increased from 0 to 24 h under salinity stress, while the target steeply decreased relative to the tolerant genotype. Further sequence analysis of the two genotypes indicated that the significant expression difference was due to the difference of methylation in the promoter region of osa-miR393a. The expression pattern of miR393a in our study was similar to that of osa-miR393a, which sharply increased in salinity-sensitive genotype A9 relative to tolerant B5 in both 150-Mm and 250-mM NaCl conditions (Figure 3). The osa-miR393a has been reported to negatively regulate the salinity stress tolerance in rice by repressing the auxin signaling pathway and other mechanisms [49]. Usually, stress induces miRNAs to downregulate their target mRNA [45], and the reciprocal relationship between the expression of osa-miR393a and TIR1 exists at different time points during salt stress [17,55]. In our result, the target of miR393a was a TIR1-like protein which played an important role in abiotic stress response. Compared to salinity-tolerant genotype B5, the high expression of miR393a in genotype A9 might lead to a large down-regulation of TIR1 and then weak tolerance to salinity stress. We would therefore predict another six miRNAs, which showed similar expression trends, like miR393a, between the two genotypes, and may also share the similar mechanism in response to salinity. The largely accumulation of negative regulators led to a significant decrease of their targets in the stress-sensitive genotype, which could not provide enough essential transcription factors, growth factors or other related molecules in normal metabolism and growth. However, the hypothesis needs further confirmation. Our result displayed the different responsive degrees of salinity-related miRNAs in different salinity tolerant genotypes of wild emmer, indicating that the related miRNAs played critical roles in response to salinity. From another perspective, the results suggested that the different levels of these regulators during the stress process might contribute to and partly explain the genetic variability of wild emmer. Moreover, the study also provided useful information for further comparison analysis of different salinity-tolerant genotypes of wild emmer at the molecular and genomic level. With the increased understanding of miRNAs and their roles during stress, further use of miRNA-mediated gene regulation to enhance plant stress tolerance will become more effective and reliable.

## 5. Conclusions

The salinity stress-responsive miRNAs in wild emmer were systematically identified using high-throughput deep sequencing technology in this study. Finally, 88 conserved miRNAs and 134 novel miRNAs were identified, of which 50 miRNAs were found to be salt-responsive. The qRT-PCR analysis of several representative miRNAs validated the sequencing results and revealed the expression patterns of salinity responsive miRNAs with different genotypes, stress levels and time courses. These results showed that salinity stress-responsive miRNAs present a dynamic expression variation during salinity stress, suggesting they played the vital roles in regulating the biological process of salinity response and tolerance in wild emmer and could be considered as the candidates for further functional studies. This study enriched the miRNA genetic information and resources for wild emmer wheat, which will not only contribute to a better understanding the role of miRNA in post transcriptional regulation of salinity stress response, but also facilitate the miRNA-based genetic improvement of salinity tolerance in cultivated wheat and beyond.

## Figures and Tables

**Table 1:** Summary of small RNAs (sRNAs) sequencing datasets. Ck: control, S: salt.

	S1	S2	S_Total		Ck1	Ck2	Ck_Total	
	Reads	Reads	Reads	%	Reads	Reads	Reads	%
Total reads	16,267,700	18,193,591	34,461,291	100	17,162,819	15,876,321	33,039,140	100
High quality (size ≥ 18 nt)	11,906,016	16,651,061	28,557,077	82.87	14,783,969	13,438,116	28,222,085	85.42
Redundancy (%)				80.65				83.73
Total of perfectly matched	7,594,637	9,486,577	17,081,214	59.81	5,470,601	5,799,131	11,269,732	39.93
Unique reads	2,487,193	3,093,686	5,580,879	19.54	2,117,167	2,448,167	4,565,334	16.18
Unique reads matched	1,035,449	1,066,097	2,101,546	37.66	369,710	558,073	927,783	20.32

**Table 2:** Distribution of sRNAs among different categories in wild emmer wheat.

Class	Unique Reads	%	Total Reads	%
Total reads	2,243,768	100	28,349,876	100
rRNAa	135,117	6.02	5,574,737	19.66
tRNA	27,037	1.20	1,392,454	4.91
snoRNA	4094	0.18	52,837	0.19
Other	14,361	0.64	276,293	0.97
High repeats (matched genome times >20)	95,407	4.25	3,189,419	11.25
Low expression (sequence times = 1)	1,550,607	69.11	1,550,607	5.47
Total Potential miRNA	417,145	18.59	16,313,529	57.54

rRNA: ribosomal RNA; tRNA: transfer RNA; snoRNA: small nucleolar RNA.

**Table 3:** Different expressed miRNAs in saline and normal conditions in wild emmer wheat.

Name	Sequence	Length	*p*-Value	Ratio	Mark
miR-1120a	TTCCGTCTCATAATATAAGAA	21	0.000630604	13.3564	UP
miR-1432b	ATCAGGAGAGATGACACCGAC	21	0.001282166	0.89426	DOWN
miR-160b	TGCCTGGCTCCCTGAATGCCA	21	0.000299524	13.2333	UP
miR-166b	TCGGACCAGGCTTCAATCCCT	21	0.016126572	9.08411	UP
miR-168	CCCGCCTTGCACCAAGTGA	19	7.46 × 10^−7^	0.12191	DOWN
miR-169e	GGCAGTCTCCTTGGCTAGC	19	0.000734617	0.74931	DOWN
miR-169g	GGCAAGTCCGTCCTTGGCTAC	21	0.006909309	1.13138	UP
miR-169i	CAGCCAAGGATGACTTGCCGA	21	0.036371524	1.19995	UP
miR-169j	CAAGTTGTTCTTGGCTAGC	19	0.015899833	1.0635	UP
miR-169k	TGGGCAAGTCACCCTGGCTACC	22	0.001990317	0.90879	DOWN
miR-171a	TGAGCCGAACCAATATCACTC	21	0.00400738	1.04654	UP
miR-171b	TGATTGAGCCGTGCCAATATC	21	0.008975612	1.13702	UP
miR-172	AGAATCTTGATGATGCTGCAT	21	0.001014298	14.092	UP
miR-393a	TTCCAAAGGGATCGCATTGAT	21	0.013664601	11.4734	UP
miR-393b	TCCAAAGGGATCGCATTGATC	21	0.016875444	9.49652	UP
miR-395	TGAAGTGTTTGGGGGAACTC	20	0.005497432	8.12411	UP
miR-396c	TTCCACAGCTTTCTTGAACTG	21	1.35 × 10^−7^	0.29308	DOWN
miR-398a	TGTTTTCCCAGGTCACCCCTT	21	0.03744688	4.247	UP
miR-398b	TGTGTTCTCAGGTCACCCCTT	21	0.010706403	7.30687	UP
miR-5175a	TTCCAAATTACTCGTCGTGGT	21	0.020159266	4.5196	UP
miR-9674d	ATAGCATCATCCATCCTGCCA	21	0.005919157	10.5192	UP
miR-9674f	GTAGGATGGCTGGTGCTATGG	21	0.003070376	0.83586	DOWN
miR-9772	TGAGATGAGATTACCCCATAC	21	0.008646577	9.88001	UP
Novel-102	CGCTGTCTCCGACATGGACC	20	0.000307346	0.27861	DOWN
Novel-108	TCAGGAAGGACCGCATCATC	20	0.043754142	0.84898	DOWN
Novel-110	AGTTTTTTCTACGACACTTTAGATTCT	27	0.04597715	7.5706	UP
Novel-111	GTCTCTGCCAATTCTTCGTGT	21	0.02294524	0.68635	DOWN
Novel-112	TCAGGAAGGACTGCATTATC	20	0.010667151	0.60942	DOWN
Novel-113	CCCGCTGTCTCCGACATG	18	0.00241642	0.37701	DOWN
Novel-125	TGCCTTTAAGGCACCTGCCTTT	22	0.011360968	7.19148	UP
Novel-14	ACAACGTTACAAAGAACT	18	0.011088055	6.70541	UP
Novel-20	CTCTCCTGTAGAAATAGGCACCGA	24	0.044368782	5.26639	UP
Novel-25	TTCCGTCTCATAATATAAGAA	21	0.014815363	13.3564	UP
Novel-27	GTGTTCTCAGGTCGCCCCCGC	21	0.006439364	0.5309	DOWN
Novel-28a	ACAAGATATTGGGTATTTCTGTCTTTATT	29	0.012777427	6.47692	UP
Novel-28b	AACAAGATATTGGGTATTTCTGTCTTTATT	30	0.000878252	33.9326	UP
Novel-3	TACGCAGAGTGAATCGGTC	19	0.000906629	0.27046	DOWN
Novel-38	TAGCGAAATTCCTTGTCGGGT	21	0.004373032	0.45084	DOWN
Novel-4	CGAGGACCTTGGTTGAGCCTG	21	0.002502204	7.85936	UP
Novel-40	TAGTAGCACCTTAGGATGGCATA	23	0.014233197	5.35626	UP
Novel-41	TATCCTCGTCGTATTCTTTATA	22	0.010294018	21.6221	UP
Novel-44	AGTAATTTTGGACGGAGGGAG	21	0.005345682	4.99639	UP
Novel-48	TATTTGTTTGCAGAGGGAGTA	21	0.019775829	4.616	UP
Novel-55	ATAAACCGGGTTTTCTGAAGCACC	24	0.035691617	0.64187	DOWN
Novel-57	TCATTTGGAACTCGCCGGTGC	21	0.002525248	0.43535	DOWN
Novel-66	CCCATGGATTGGCTAGTTCCT	21	0.04578047	4.32044	UP
Novel-74	TGCATCATTTGGAACTCGCC	20	0.015178025	0.72326	DOWN
Novel-87	AAATGGAATTTAACTCTTTCATGCT	25	0.039866375	4.0404	UP
Novel-92	AGCCAACAACCTCCTAGTTCC	21	0.018601675	0.75506	DOWN
Novel-99	ATGCCGTGTTGTTCTGAAAGAA	22	0.024139855	9.38665	UP

Note: UP means up-regulated expression under salt stress compared to normal condition; DOWN means down-regulated expression under salt stress compared to normal condition.

**Table 4:** Salinity and drought miRNAs in wild emmer and related species.

	*Triticum dicoccoides*		*Aegilops tauschii*		*Triticum aestivum*		*Hordeum Vulgare*		*Brachypodium distachyon*		Putative Target
	D	S	D	S	D	S	D	S	D	S	
miR1074						UP					Malate dehydrogenase
miR1120		UP									
miR1134						UP					
miR1432	UP	DOWN									Calcium binding or Calcium ATPases
miR1435	UP										
miR1450	UP								UP		Mn superoxide dismutase
miR156	UP				UP	UP	UP	UP	UP		SPL
miR159						UP			UP		MYB65/MYB33
miR160		UP			UP	DOWN					Metal transporter NRAMP2-like
miR164						UP/DOWN			UP		NAC TF, Got1/Sft2-like, NAM
miR166	DOWN	UP			UP		UP/DOWN				HD-Zip TF
miR167			UP		UP/DOWN	DOWN			UP		Auxin response factor
miR168		DOWN						DOWN			CCAAT-binding TF
miR169		UP				DOWN			UP		Polyol transporter 5-like
miR171	DOWN	UP			UP	UP/DOWN	UP/DOWN	UP			Auxin response factor
miR172		UP			DOWN	DOWN					Argonaute/AP2/EREBP TF
miR1867	UP										DUF1242 superfamily
miR1881	UP								UP		
miR319						DOWN			DOWN		TCP TF21
miR3440						DOWN					
miR3710						UP					VAMP protein SEC22
miR393		UP				UP					auxin F-box
miR395		UP			UP	DOWN			UP		ATP sulfurylase, APS4/F-box
miR396	DOWN	DOWN			UP	DOWN			UP		GRF1/GRF2, GRF2 & SYP131
miR398	Up	UP									programmed cell death protein 4
miR408					UP						Laccase
miR444						UP	DOWN	DOWN			MADS-box TF 27-like
miR474	UP										Kinesin, PPR family
miR482					UP	UP					Rab15B protein
miR5021						UP					
miR5024	UP										
miR5049				D-E		UP					60S ribosomal protein l36
miR5083						DOWN					
miR5175		UP	DOWN		UP						ACC-like oxidase
miR5205			DOWN	D-E	DOWN						
miR5227						UP					6-phosphofructokinase 2-like
miR5266						DOWN					
miR528	DOWN				DOWN				UP/DOWN		XBAT32
miR529						UP					E3 ubiquitin ligase RNF14-like/bHLH TF
miR535						UP/DOWN					5.8S ribosomal RNA gene
miR5523			DOWN								
miR5568				D-E							
miR5655						UP					TF bHLH135-like
miR6108						UP					Alpha tubulin
miR6220				D-E							
miR6222						DOWN					
miR6248					UP						
miR7714	UP										
miR894	UP										Zinc finger and C2 domain protein-like
miR896	DOWN										
miR903						DOWN					26S ribosomal RNA gene
miR9674		UP/DOWN									
miR9772		UP									

In the table the different expression patterns of miRNAs under salt and drought are marked as induced (UP) or repressed (DOWN) expression. D-E: Differently expressed. D: drought; S: salt. References—*Triticum turgidum* ssp. *dicoccoides*: [22,23] *Aegilops tauschii*: [43,51] *Triticum aestivum*: [20,39,44,45,52] *Hordeum vulgare*: [7,53] *Brachypodium distachyon*: [47,54].

## Supplementary Materials

The following are available online at www.mdpi.com/2073-4425/8/6/156/s1. Figure S1: (A) Length distribution of sequencing reads (nt); (B) Length distribution of unique reads (nt), Figure S2: (A) Nucleotide bias at different sites from 1 to 24 nt (5′–3′); (B) First nucleotide bias for different lengths of 18–24 nt, Figure S3: Predicted hairpin structures of novel miRNA precursors, Figure S4: Relative expression levels of the validated miRNAs by stem-loop qRT-PCR, Table S1: Conserved and novel miRNA identified in wild emmer by deep sequencing, Table S2: List of putative target genes of miRNAs in wild emmer, Table S3: Primers used in qRT-PCR, Table S4: GO annotation of the total targets genes of miRNAs in wild emmer, Table S5: KEGG analysis of the total targets genes of miRNAs in wild emmer, Table S6: GO annotation of the total targets genes of salinity responsive miRNAs in wild emmer, Table S7: KEGG analysis of the total targets genes of salinity-responsive miRNAs in wild emmer.
