# Supplementary material for: Genome-Wide Identification and Characterization of Salinity Stress-Responsive miRNAs in Wild Emmer Wheat (Triticum turgidum ssp. dicoccoides)
Source: Genes (Basel). 2017 Jun 6;8(6):156. doi: 10.3390/genes8060156 (PMC5485520; doi:10.3390/genes8060156)
Supplement: Supplementary file 1 [file genes-08-00156-s001.zip › Supplementary_revised/Figure S4.pdf]

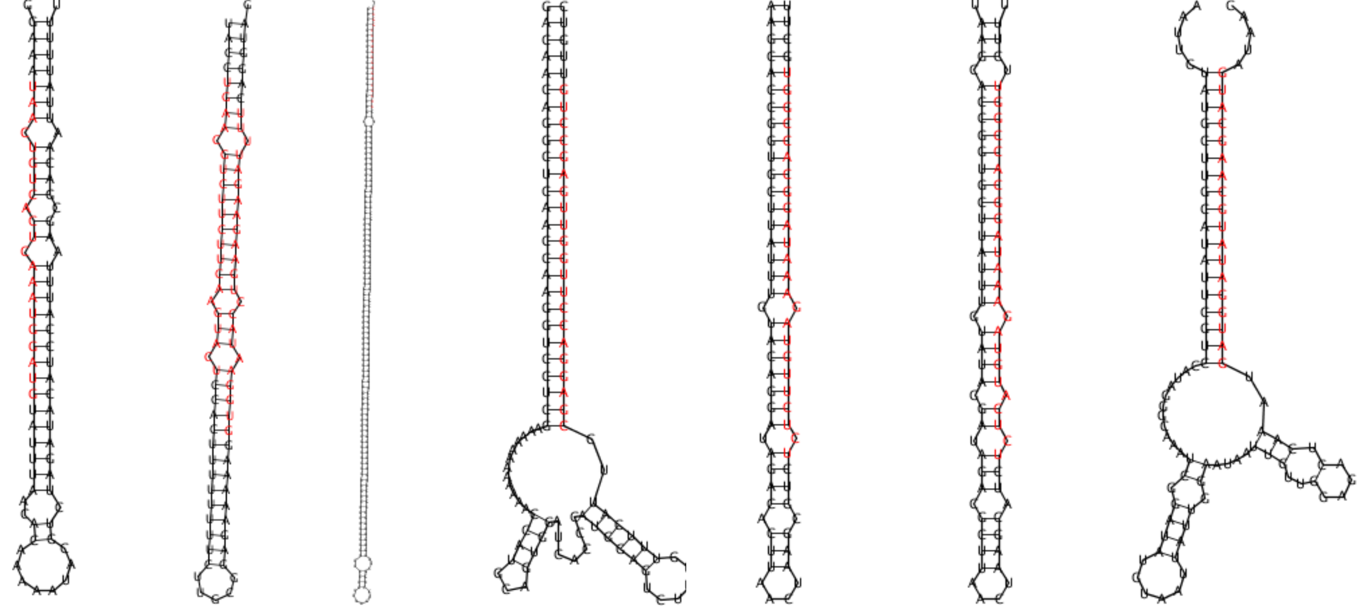

Novel-1 Novel-2 Novel-3 Novel-4 Novel-5a Novel-5b Novel-6

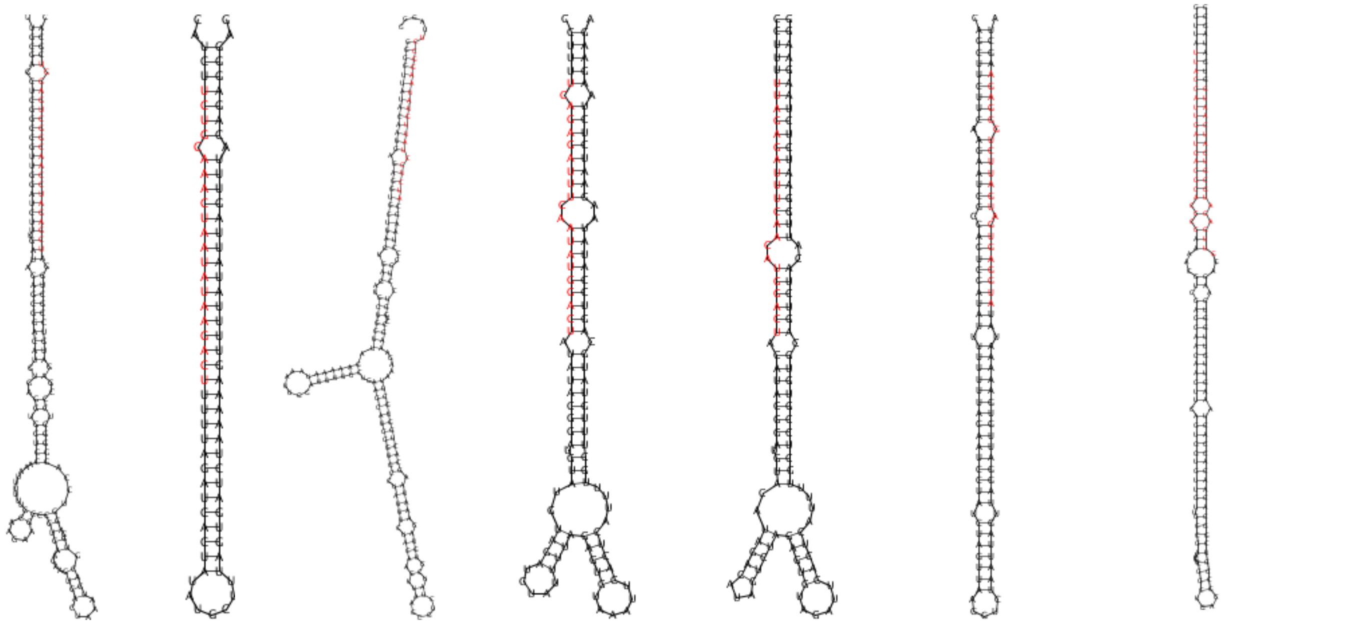

Novel-7 Novel-8 Novel-9 Novel-10a Novel-10b Novel-11 Novel-12

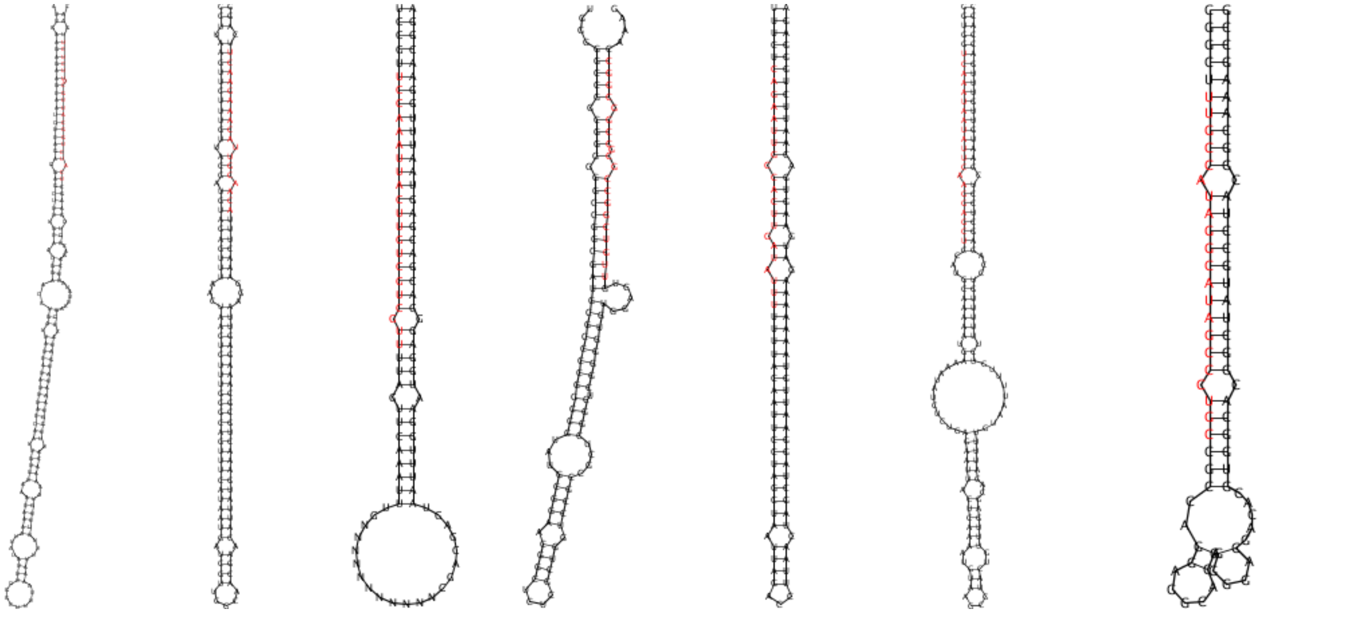

Novel-13 Novel-14 Novel-15 Novel-16 Novel-17 Novel-18 Novel-19a

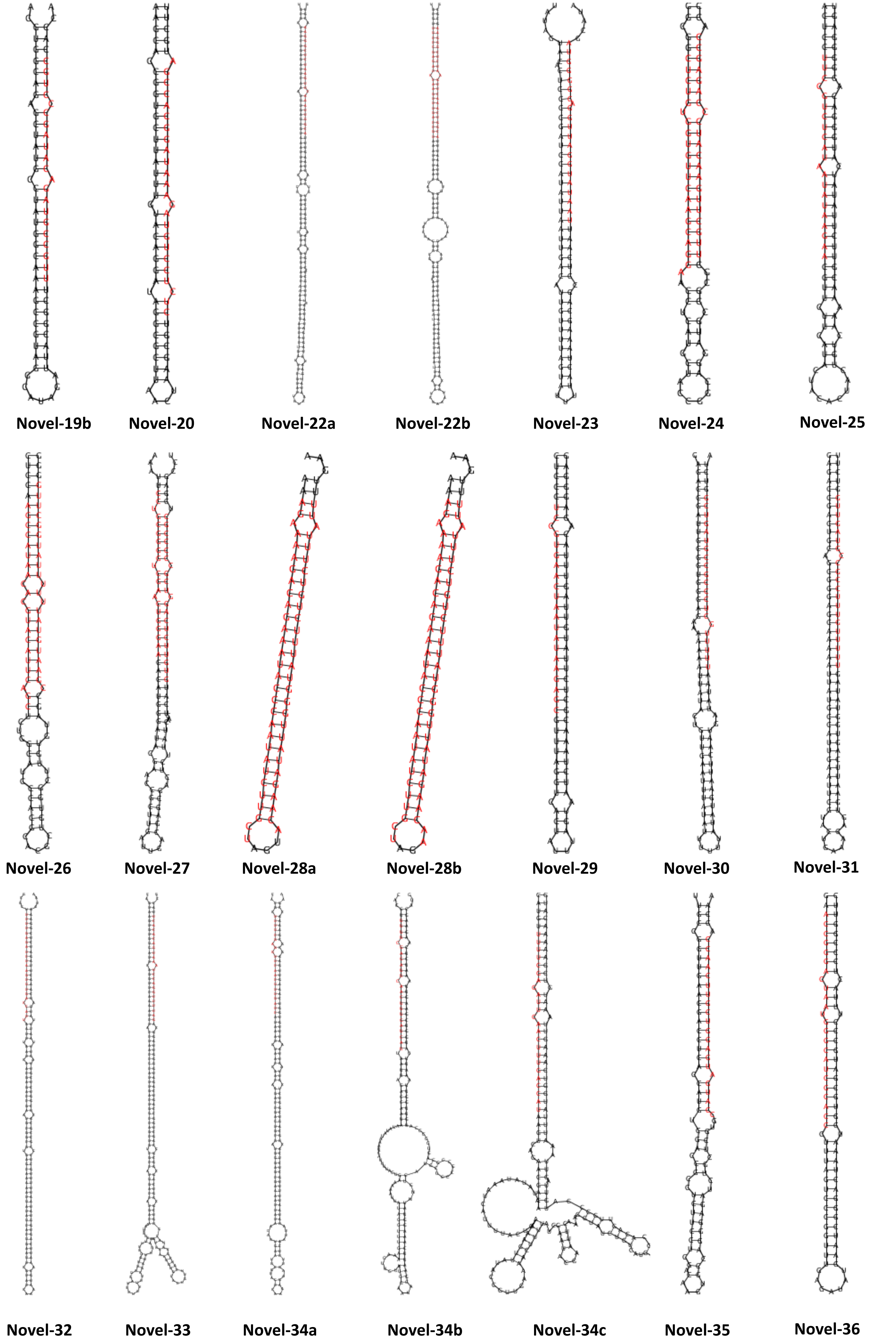
Novel-19b
Novel-20
Novel-22a
Novel-22b
Novel-23
Novel-24
Novel-25
Novel-26
Novel-27
Novel-28a
Novel-28b
Novel-29
Novel-30
Novel-31
Novel-32
Novel-33
Novel-34a
Novel-34b
Novel-34c
Novel-35
Novel-36

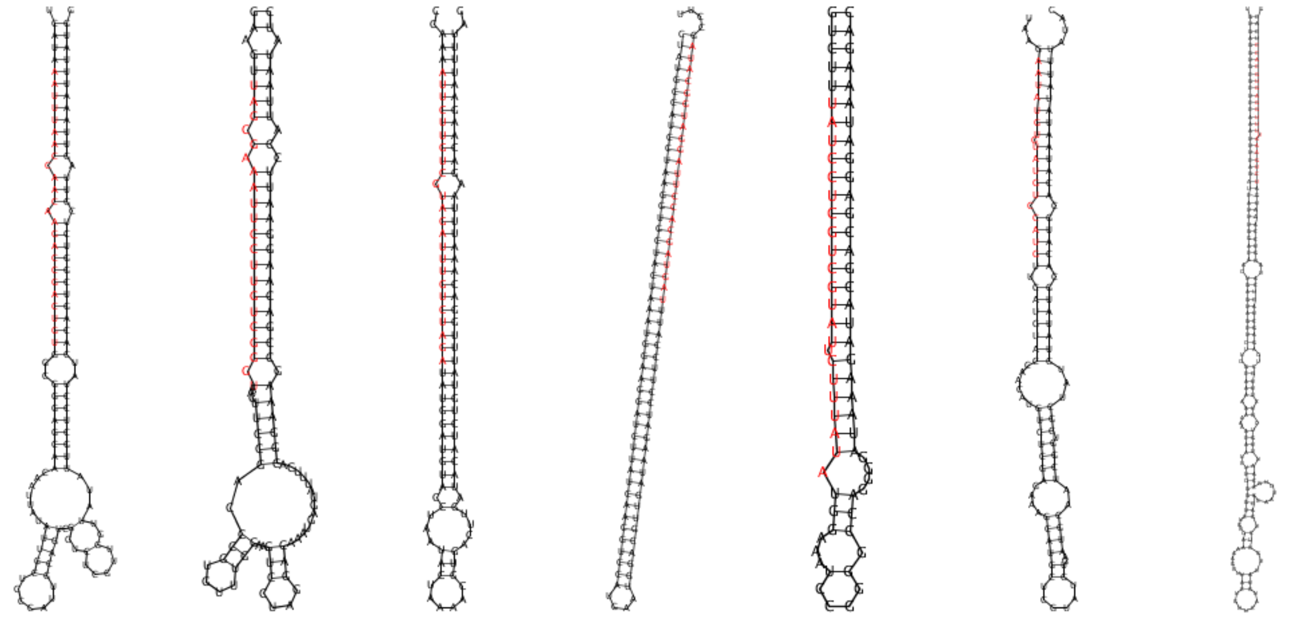

Novel-37 Novel-38 Novel-39 Novel-40 Novel-41 Novel-42 Novel-43

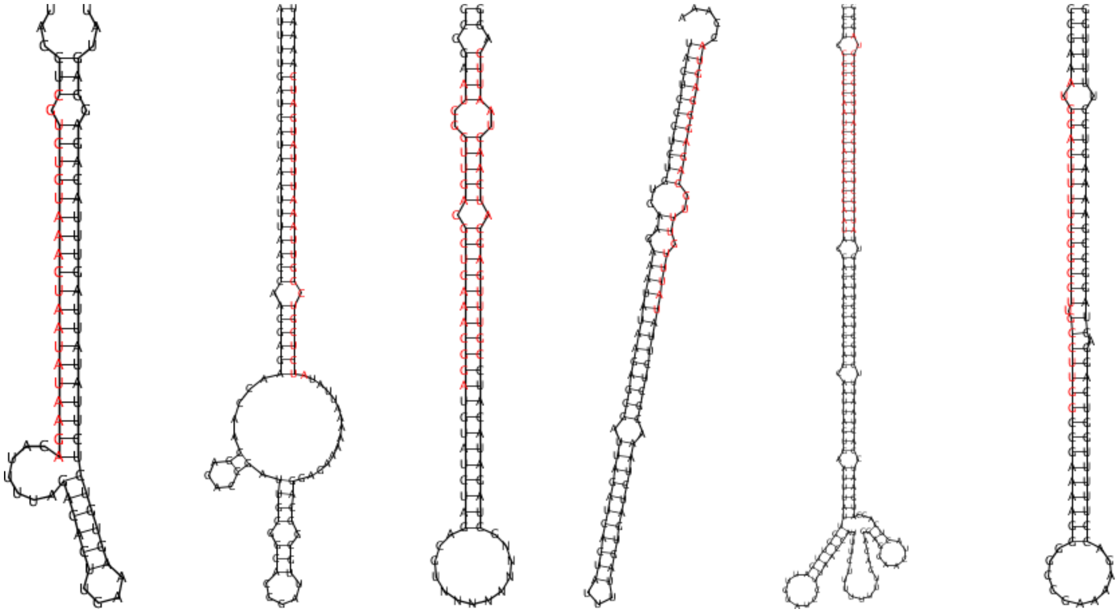

Novel-44 Novel-45 Novel-46 Novel-47 Novel-48 Novel-49 Novel-50

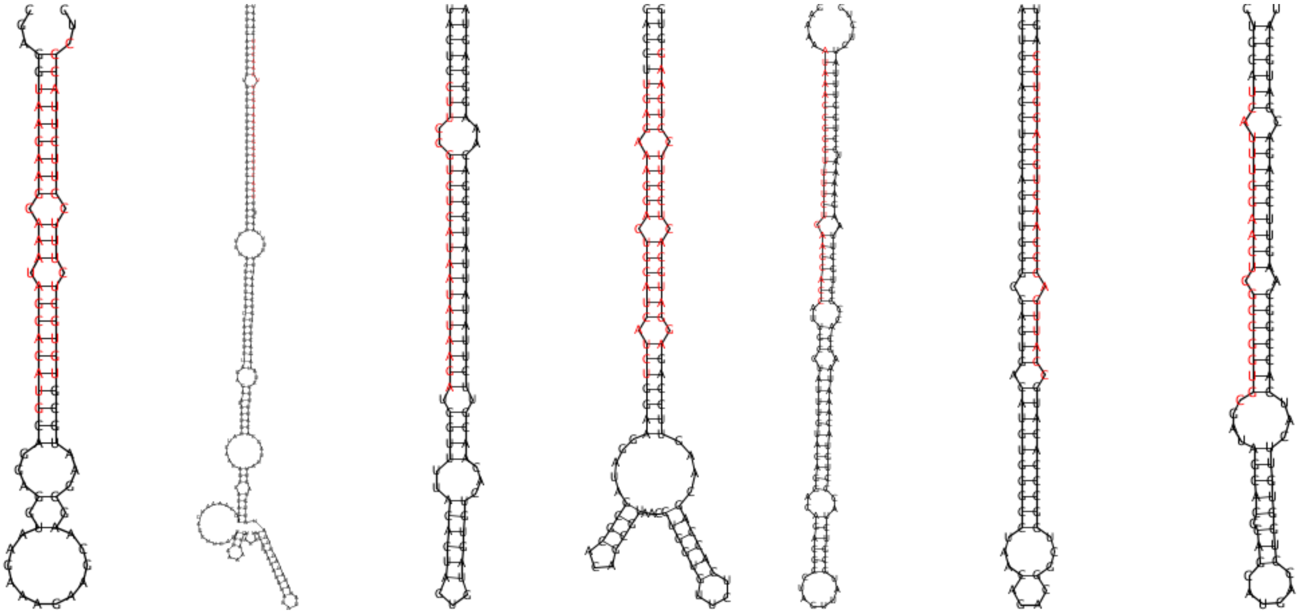

Novel-51 Novel-52 Novel-53 Novel-54 Novel-55 Novel-56 Novel-57

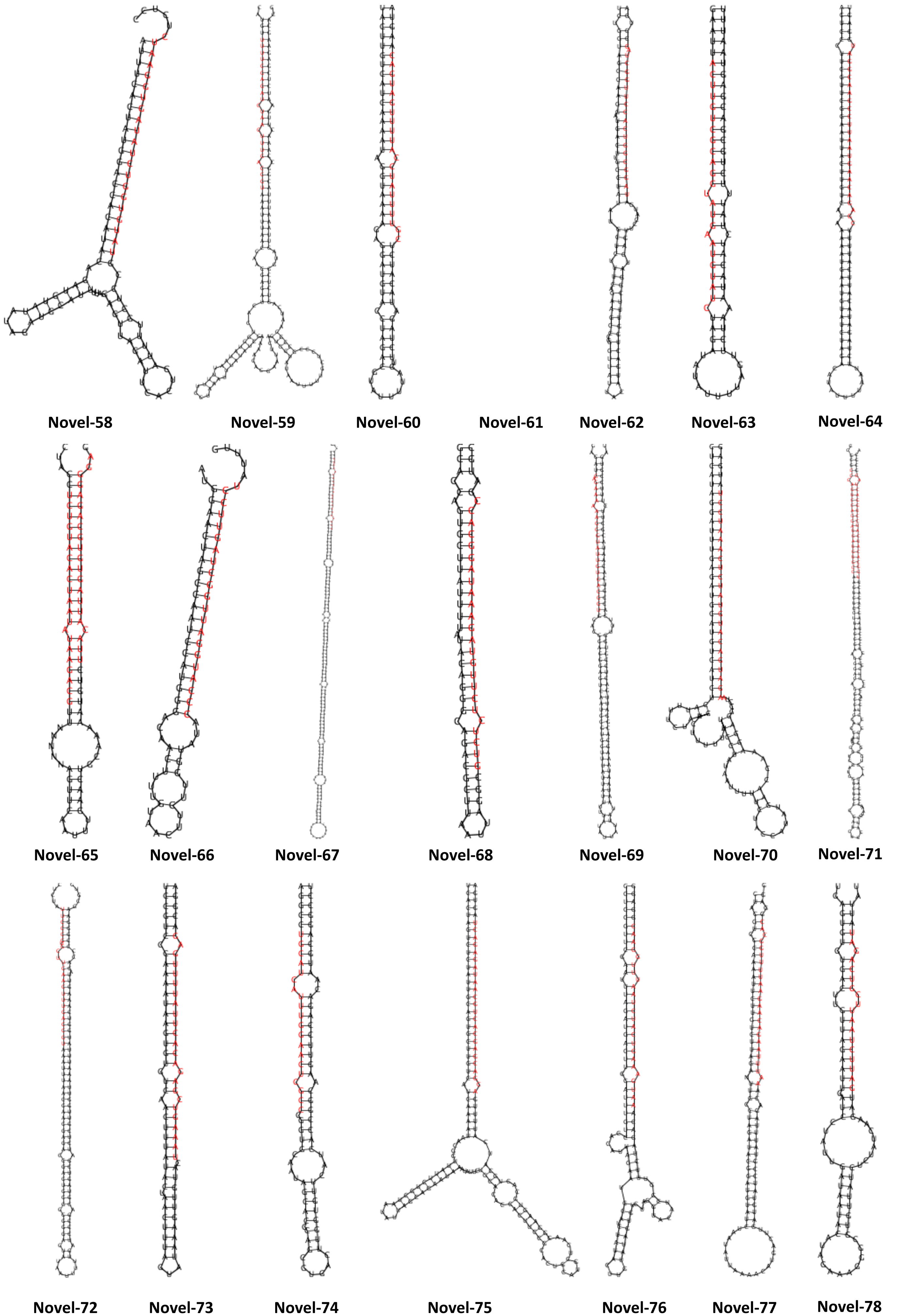

Novel-58
Novel-59
Novel-60
Novel-61
Novel-62
Novel-63
Novel-64
Novel-65
Novel-66
Novel-67
Novel-68
Novel-69
Novel-70
Novel-71
Novel-72
Novel-73
Novel-74
Novel-75
Novel-76
Novel-77
Novel-78

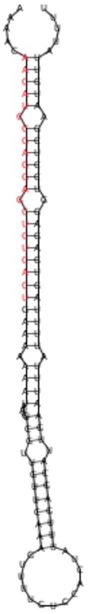
Novel-79

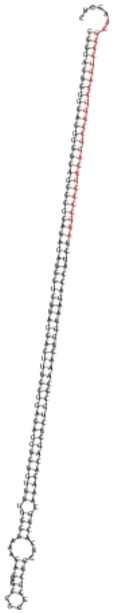
Novel-80

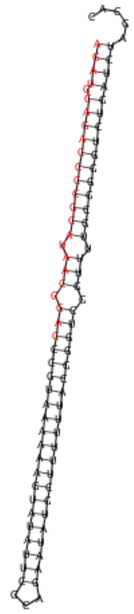
Novel-81

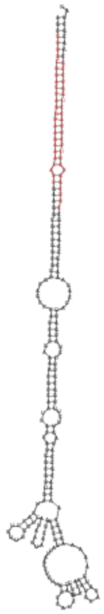
Novel-82

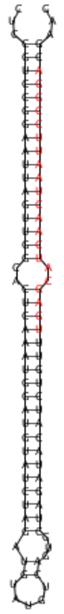
Novel-83

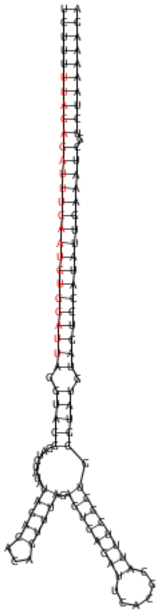
Novel-84

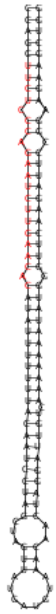
Novel-85

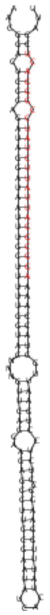
Novel-86

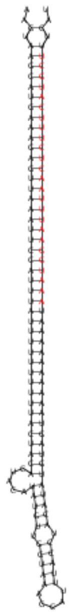
Novel-87

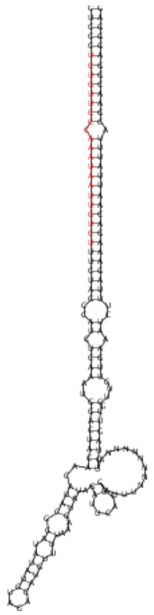
Novel-88

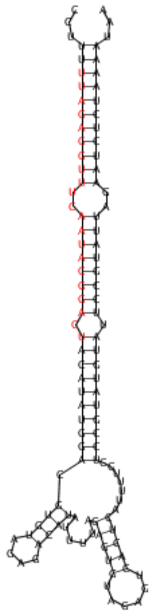
Novel-89

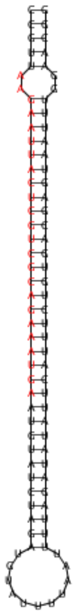
Novel-90

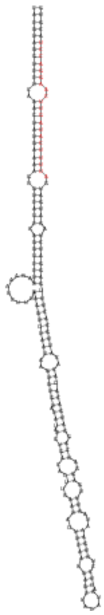
Novel-91

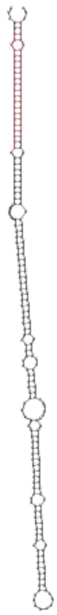
Novel-92

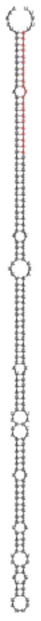
Novel-93

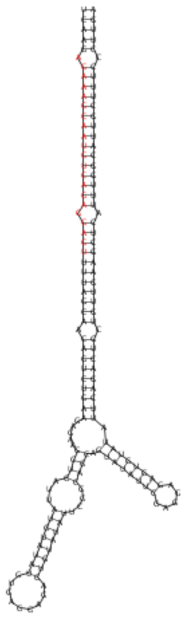
Novel-94

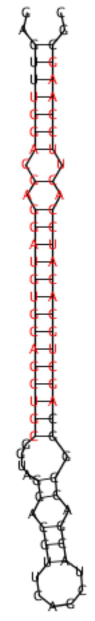
Novel-95

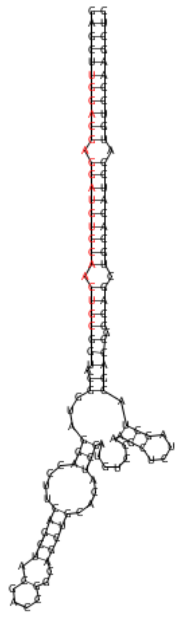
Novel-96

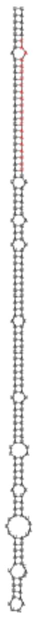
Novel-97

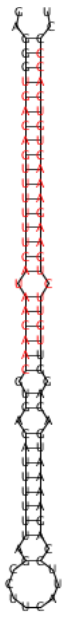
Novel-98

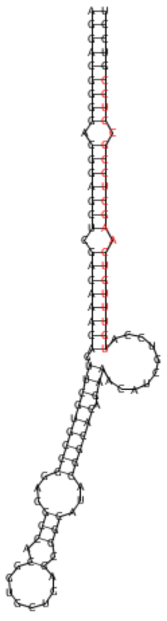
Novel-99

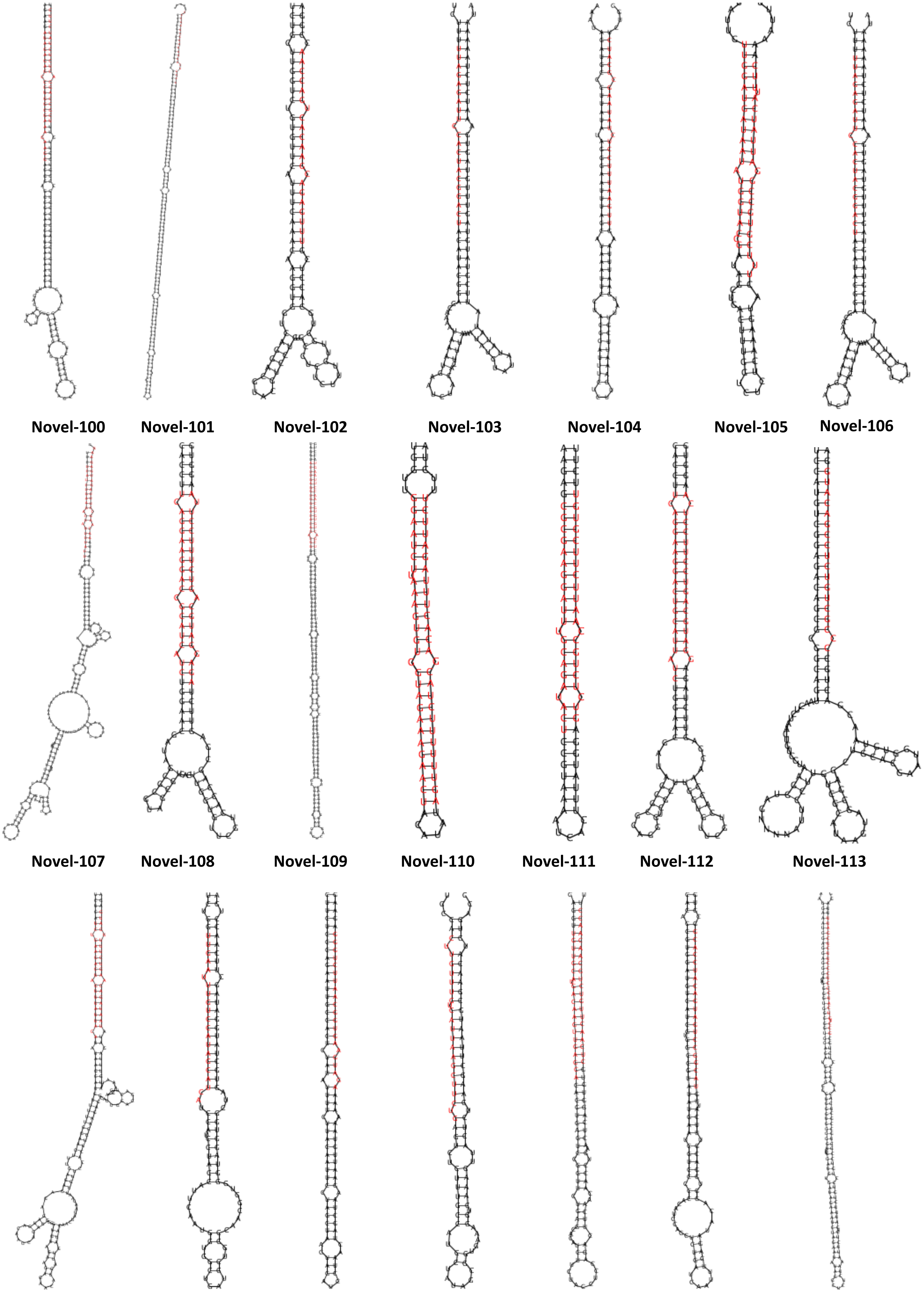
Novel-100
Novel-101
Novel-102
Novel-103
Novel-104
Novel-105
Novel-106
Novel-107
Novel-108
Novel-109
Novel-110
Novel-111
Novel-112
Novel-113
Novel-114
Novel-115
Novel-116
Novel-117
Novel-118
Novel-119
Novel-120

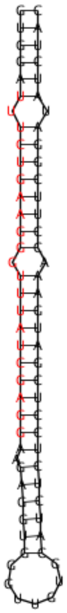

Novel-121

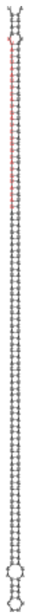

Novel-122

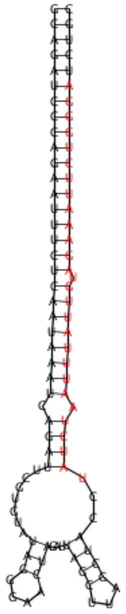

Novel-123

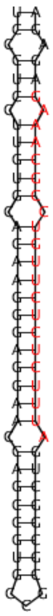

Novel-121

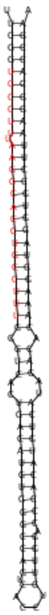

Novel-122

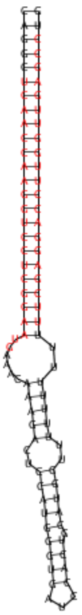

Novel-123
