# Supplementary figures and images for: Genome-Wide Identification and Characterization of Salinity Stress-Responsive miRNAs in Wild Emmer Wheat (Triticum turgidum ssp. dicoccoides)
Source: Genes (Basel). 2017 Jun 6;8(6):156. doi: 10.3390/genes8060156 (PMC5485520; doi:10.3390/genes8060156)

A

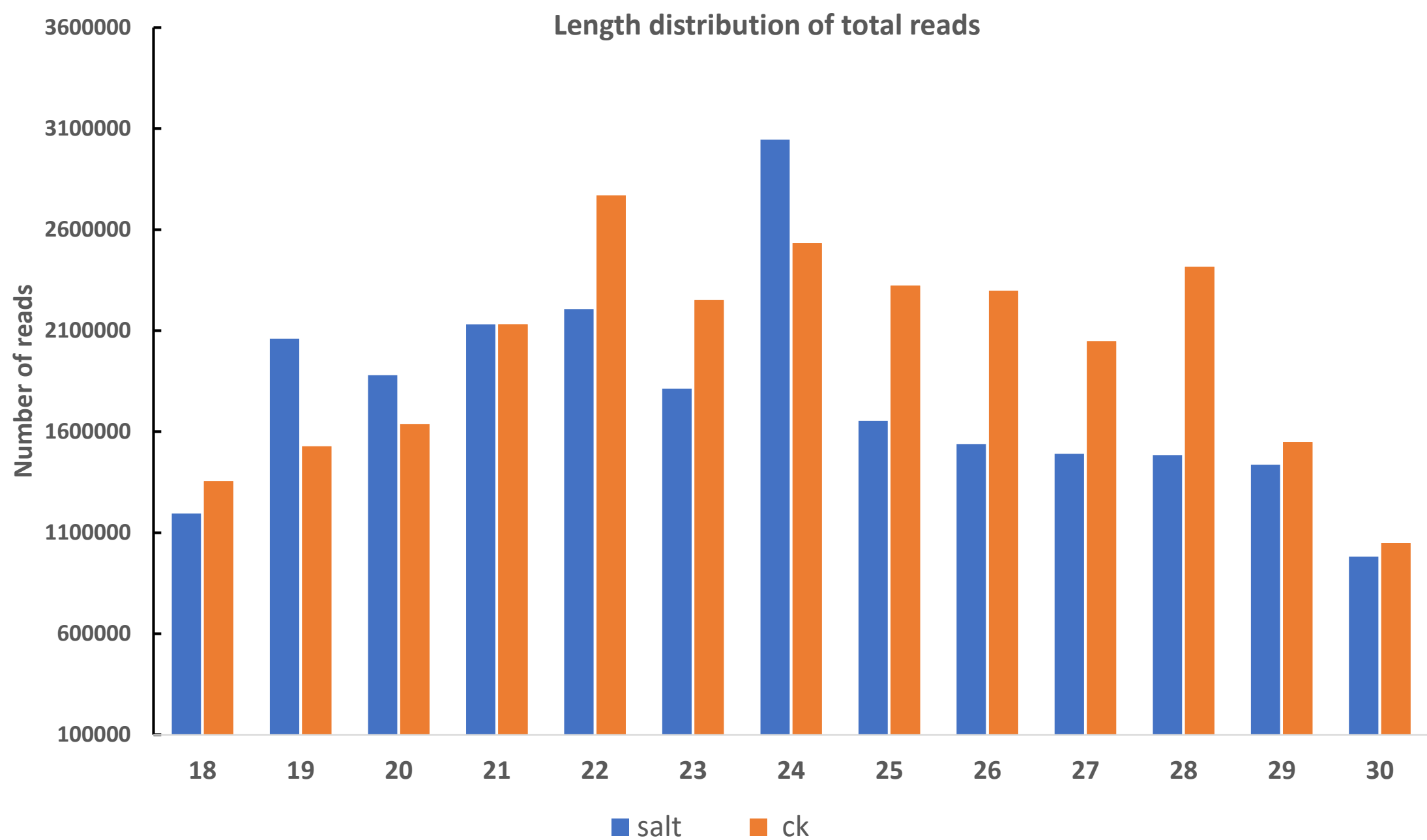

B

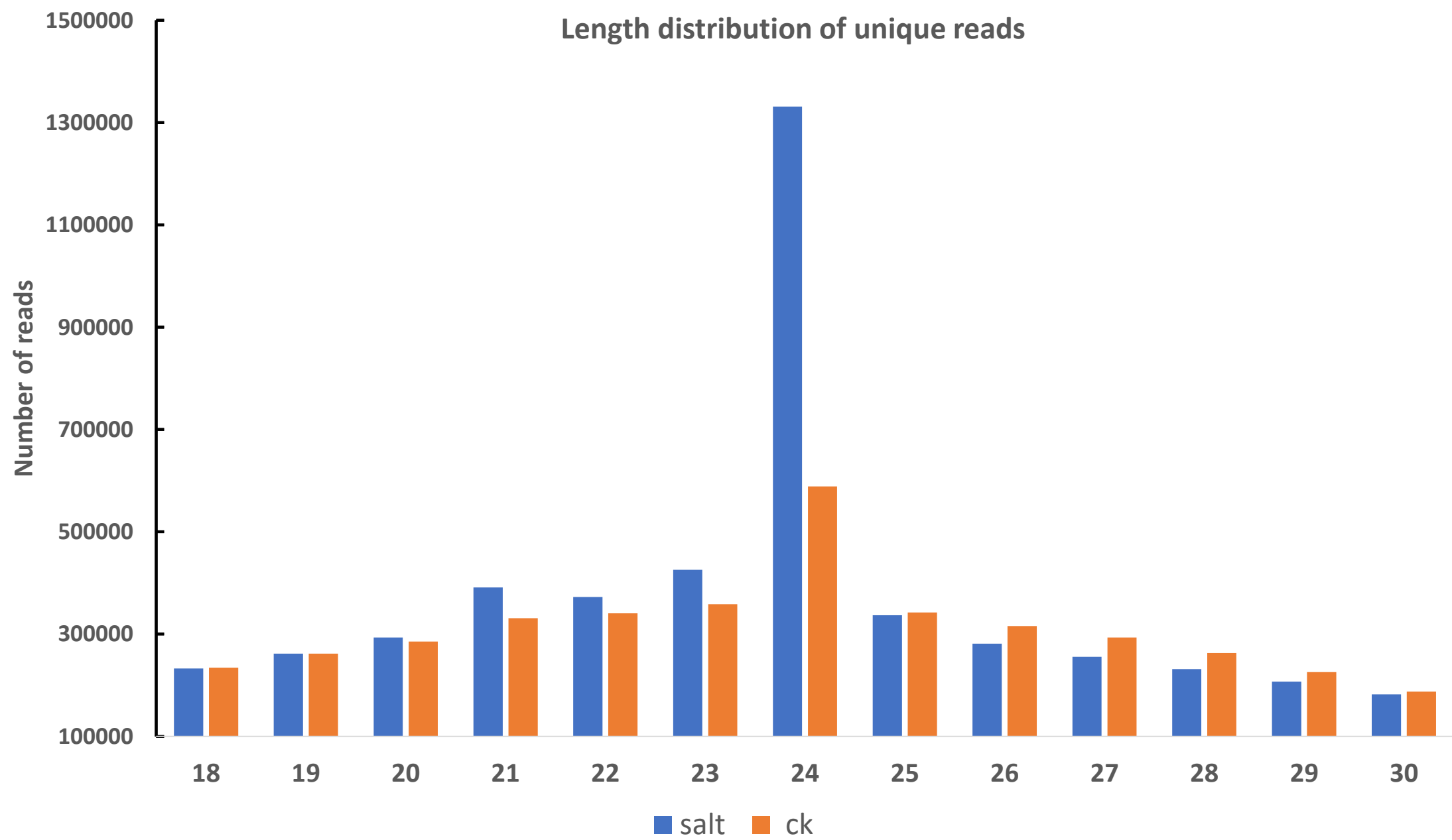

Supplement: Supplementary file 1 [file genes-08-00156-s001.zip › Supplementary_revised/Figure S1.pdf]

A

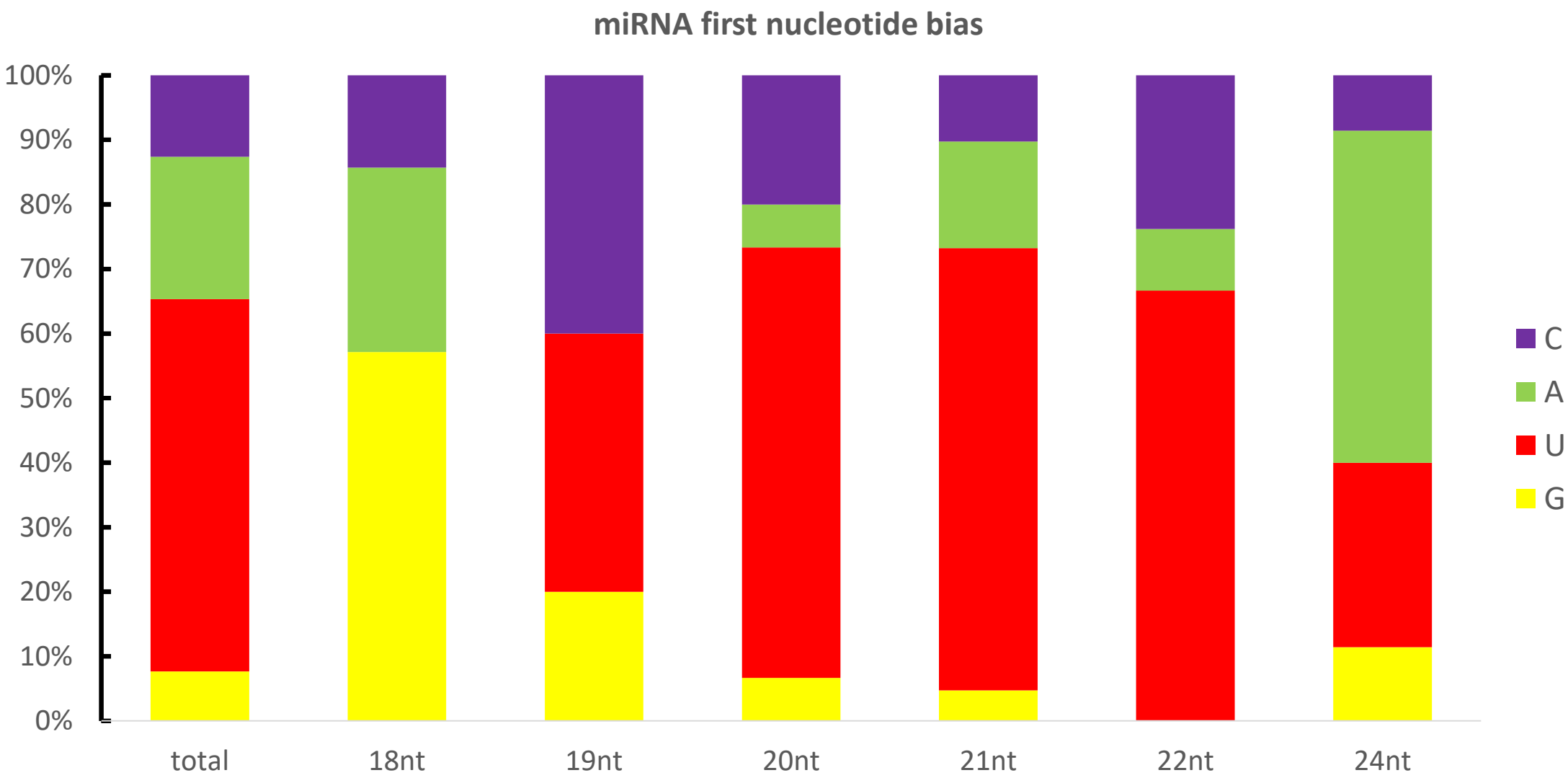

B

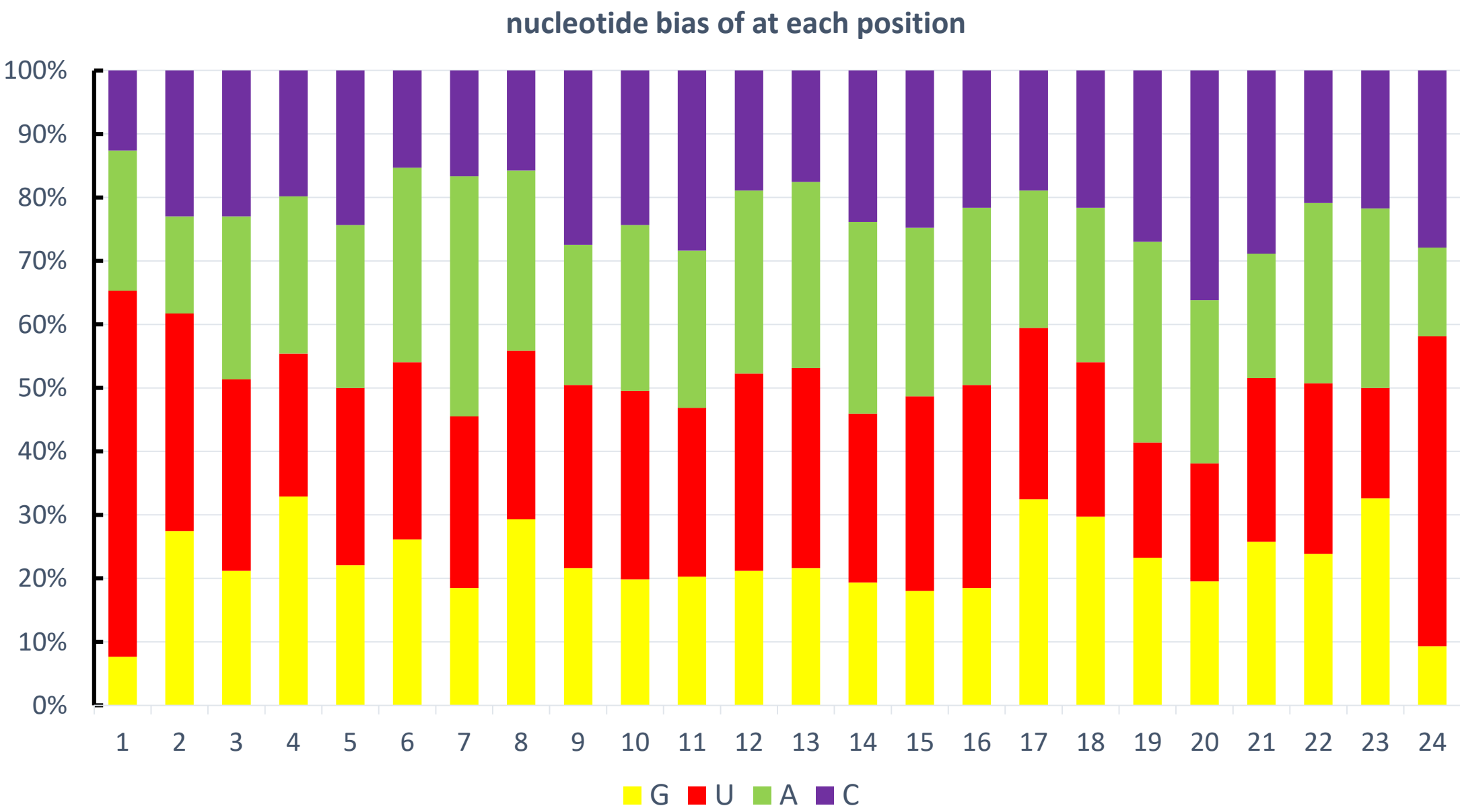

Supplement: Supplementary file 1 [file genes-08-00156-s001.zip › Supplementary_revised/Figure S2.pdf]

Relative Expression Ratio

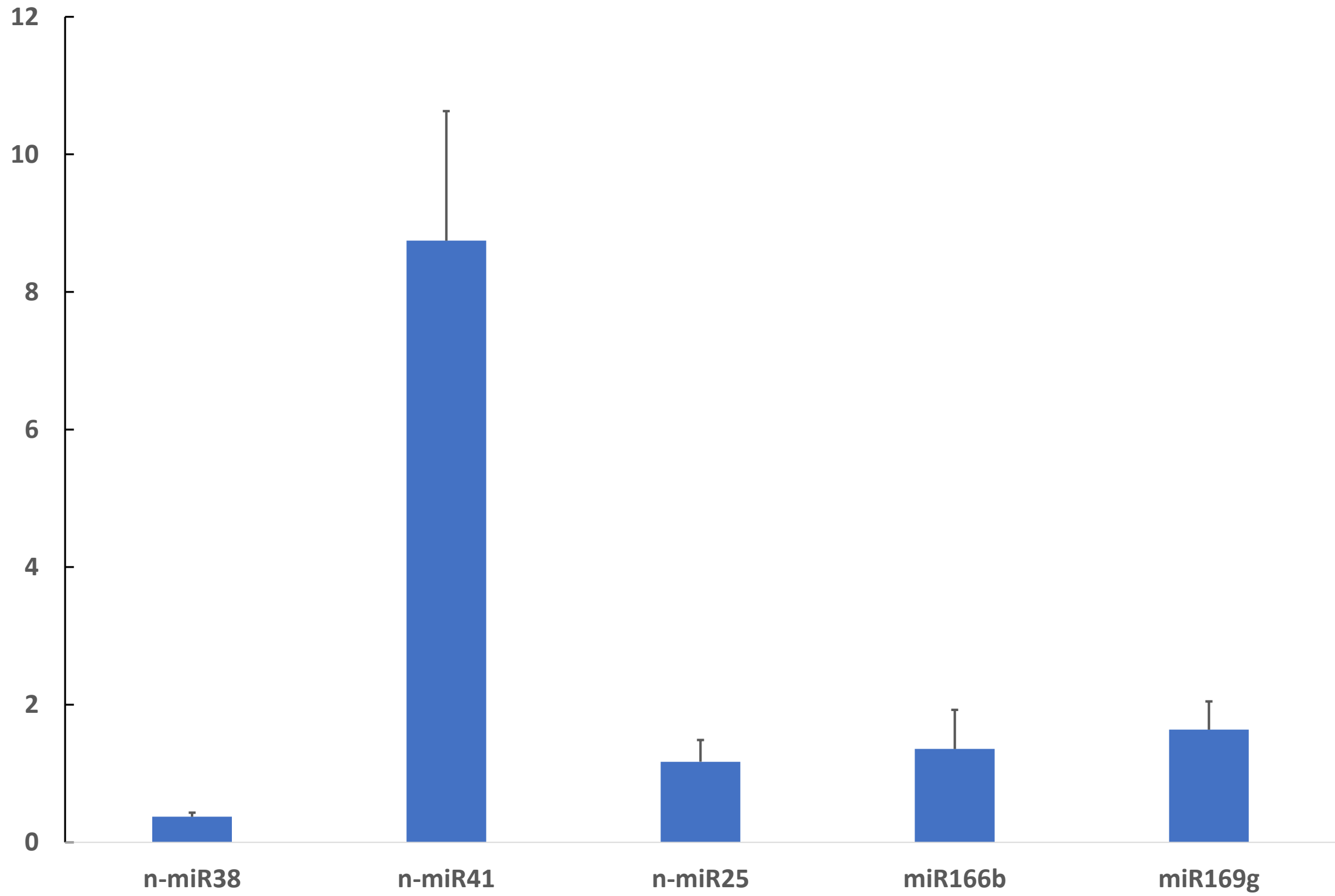

Supplement: Supplementary file 1 [file genes-08-00156-s001.zip › Supplementary_revised/Figure S3.pdf]
